# Composition of place, minority vs. majority group-status, & contextualized experience: The role of level of group representation, perceiving place in group-based terms, and sense of belonging in shaping collective behavior

**DOI:** 10.1371/journal.pone.0253571

**Published:** 2021-09-20

**Authors:** Demis E. Glasford

**Affiliations:** John Jay College and Graduate Center, The City University of New York (CUNY), New York, New York, United States of America; Rice University, UNITED STATES

## Abstract

The current studies (N = 1,709) explore why demographic composition of place matters. First, this work demonstrates that relative level of group representation affects one’s experience of place in the form of self-definition (*self-categorization*), perceptions of place being representative or characteristic of factors that distinguish the group from others (*place-prototypicality*), and sense of belonging (*place-identification;* Studies 1a-1e; Studies 2a & 2b). Second, the studies illustrate that group representation within place shapes the way group member’s approach (i.e., expectations of group-based treatment and procedural justice; Studies 2a-2c), understand (i.e., attribution for group-based events, Study 2b; responsiveness to bias-reduction intervention, Study 4a; sense of solidarity, Study 4b), and behave (i.e., prejudice, Studies 3a & 3b; collective action, Study 4c). More broadly, I present a *S**ocial identity* *Pa**radigm for* *C**ontextualized* *E**xperience* (SPACE) that provides an organizing framework for the study of the impact of characteristics of place on social identity-based contextualized experience and (in turn) collective behavior. Taken together, the findings provide evidence of distinct psychological experience and orientation as a function of minority versus majority-group status within place, as well as for a group-based approach to place. Implications for the study of collective and intergroup behavior are discussed.

## Introduction

“Are there few or many people like me here?” is a question that becomes salient to a person almost immediately upon entering a space or place. Although all collective and intergroup behavior occurs within place, the study of *the place of intergroup relations* has historically received relatively little empirical attention within the intergroup relations literature. Recent work, however, has begun to interrogate the role of place in shaping intergroup relations [1,2]. In the present work, I argue that level of group representation within place shapes perceptions of the group-based nature of place and sense of belonging, which in turn inform numerous and a diverse set of collective and intergroup outcomes. Taken together, the evidence supports the notion that there are distinct psychological orientations as a function of low (minority-group status) versus high (majority-group status) group representation within place. More broadly, I introduce a *S**ocial identity* *Pa**radigm for* *C**ontextualized* *E**xperience* (SPACE) that provides a framework for understanding how the characteristics of place inform social identity-based contextualized experience, as manifested by three social identity constructs: self-categorization, place-prototypicality, and place-identification. Thus, the present work is concerned with one specific characteristic of place theorized to be central to shaping group member’s contextualized experience: composition of place.

## Composition of place as a determinant of social identity-based contextualized experience

Why does group representation matter? As a starting point, demographic composition of place or viewing a place as having low (i.e., numerical minority status) or high (i.e., numerical majority status) group representation relative to the total social make-up of a place, is expected to shape one’s propensity to view the respective place in group-based terms and sense of belonging. A variety of characteristics within place (taken together) likely account for a person’s contextualized experience. Thus, it is expected that numerous physical and social characteristics of a place or local context can shape group members’ experience in social identity-based terms. However, level of numeric group representation is theorized to be a foundational characteristic of place because of its capacity to affect numerous collective and intergroup outcomes.

There is a large, diverse, and interdisciplinary literature devoted to defining “place” and/or “space” [3–7], which includes a long and nuanced debate on the difference between ‘space’ and ‘place’ [8–10]. Consistent with recent work that utilizes a more expansive classification of place [2], I use a holistic definition of *place* defined as the *psychologically salient environment*, which can take the form of a geographic, social, or physical place. This definition is in line with a social identity perspective, such that psychologically salient environment is connected to frame of reference and suggests a ‘place’ definition that is established in the most applicable or relevant social identity for a set of specified intergroup relations. More generally, in the present work, I use the terms ‘*composition of place*’ and ‘*level of group representation*’ interchangeably or in an analogous/similar manner. Composition of place entails level of group representation, but also explicitly acknowledges the representation of other group(s) within place, which is an integral aspect of the factor under study. Thus, composition of place is meant to denote total demographic composition of a respective place. The current work is concerned with the degree to which one’s (in) group is represented in place, *relative* to the total composition of people within the respective place.

Places are filled with group-relevant meaning that affect how social identity informs collective behavior in the physical environment. Indeed, social identity is directly shaped by the context and place of collective or intergroup behavior, including symbols [11,12], objects of place and sense of belonging [13,14], affecting perceptions of threat [15] and evaluative judgment of out-groups [1,16,17]. The present work seeks to complement these findings by explicitly linking how one characteristic of place, the social make-up in terms of numeric group representation (e.g., having minority vs. majority-group status), changes the way a person experiences the context in social identity-based terms and (in turn) their collective and intergroup behavioral tendencies. To start, a central thesis of the present work is that degree of in-group representation of a context gives a place group-relevant meaning by shaping propensity to view the respective place in group-based terms. One way a person can view a place in group-based terms is via perceptions of the group prototypicality of place.

### A group-based approach to place: Viewing place in group-based terms & place-prototypicality

At the heart of the present work is a contrast between two distinct ways of understanding place: group neutral versus group-based. A variety of places are either assumed or implicitly designed to be group-neutral—meaning the place is not identified, characteristic, representative, or necessarily *for* (legally) any particular group. These places include classrooms (education), doctor’s office or hospitals (health), organizations (business), police precincts (law enforcement), and locker rooms (sports). Whereas a group-neutral approach to place starts with an assumption that places are not ‘of a particular group or groups,’ a group-based approach to place assumes that places are full of characteristics that provide group-relevant meaning (i.e., can be of a particular group or groups). The two approaches differ not only regarding the notion that people can and do view places in group-based terms, but also with respect to the starting point for approaching and understanding behavior of place (i.e., all groups are equal within place vs. groups differ in viewing place in group-based terms). Importantly, a group-based approach to place suggests that even places designed to be group-neutral may not necessarily be perceived as group-neutral by all groups. There are a variety of ways to view place in group-based terms, but the present work is concerned with how level of group representation affects a group member’s perceptions regarding the group-prototypicality of place.

Prototypes, and more specifically perceptions of prototypicality, are central to the social identity perspective, and therefore integral to understanding collective behavior. Prototypes are fuzzy sets of attributes that capture the features that characterize a group and distinguish the group from other groups [18]. There is evidence not only that targets vary in perceived prototypicality [e.g., leaders; 18], but also that members of groups are often more likely to be responsive to targets that are viewed as relatively more prototypical of their group, compared to targets viewed as less prototypical of their group [19]. More specifically, the extent to which leaders share group-defining qualities [20], emphasize goals that are characteristic of the group [21], or are representative of the typical demographics of the group [22], all directly influence perceptions of the prototypicality of a leader. Moreover, perceived group-prototypicality has a direct influence on how group members respond to leaders (see Haslam, Reicher, & Platow, 2011; van Knippenberg, 2011 for reviews). Indeed, variance in perceived prototypicality of a leader explains perceptions of fairness [20], trust [23], endorsement of leaders [24], and overall leader effectiveness [25]. Whereas prior theorizing on prototypicality has focused primarily on leader prototypicality [see 26,27 for reviews], the current work extends the study of group-prototypicality to place. In the present work, I extend prototypicality to place and introduce the construct of *place-prototypicality*.

Just as people can be viewed in terms of degree of group prototypicality, such that some people are viewed are more prototypical of one’s group compared to others [e.g., leaders; 27], it is expected that places can be viewed in terms of group prototypicality, such that group members perceive places as varying in degree of group prototypicality for their own group. Drawing on past empirical work [28,29], *place-prototypicality* is defined as the extent to which a given place is perceived as being characteristic of a group, representing the unique values of the group, and exemplifying the beliefs that define the group. Thus, a central assumption of this work is that people perceive places as holding a predominant set of group-based characteristics, beliefs or values. Accordingly, group-members make judgements about the group-prototypicality of place or the extent to which a place is characteristic of their own group. Consistent with work on leadership [27], it is expected that characteristics of a place should inform perceptions of place-prototypicality, but also that group members will respond more positively to places perceived as high in place-prototypicality, compared to places low in place-prototypicality. Rather than ignoring context to rely on (reductionist) individualistic and interpersonal frameworks—devoid of contextualized experience, the present work starts with the assumption that collective phenomena are best understood by factoring in characteristics of the situated context [30]. In seeking to examine how the social make-up of place shapes behavior, it is expected that perceptions of place-prototypicality are just one facet of a broader social identity-based contextualized experience that shapes collective and intergroup behavior.

### Integrating place-prototypicality into ‘social-identity based’ contextualized experience

To best understand a group member’s behavior, it is integral to account for the group member’s contextualized experience—situated and embedded within the respective place of relevant intergroup relations. For the purposes of the current work, there are three questions at the forefront of people’s experience within a given social context: “How do I think of myself in this context or place?” (*self-categorization*), “Is this place characteristic of my group?” (*place-prototypicality*), and “Do I identify with this place?” (*place-identification*). That is, that the way one defines oneself (self-categorization), one’s perception of whether a place is representative of the characteristics that make their group unique from other groups (place-prototypicality) and one’s sense of belonging (place-identification) are critical to what could be termed a person’s ‘social identity-based contextualized experience.’ More broadly, the present work seeks to link characteristics of place, social identity-based contextualized experience, and collective behavior. A *S**ocial identity* *Pa**radigm for* *C**ontextualized* *E**xperience* (SPACE) provides a framework for understanding how the characteristics of place inform social identity-based contextualized experience, which (in turn) organize how group members approach, understand, and behave within the respective place.

### A *S*ocial identity *Pa*radigm for *C*ontextualized *E*xperience (SPACE)

In line with the social identity perspective [31,32] and work rooted in situational cues of context [14,33], a SPACE framework suggests that characteristics of a place and three facets social-identity based contextualized experience (self-categorization, place-prototypicality, and place-identification) shape collective and intergroup behavior. More specifically, the three basic tenets of a SPACE approach are as follows:

1. Characteristics of place determine self-categorization, place-prototypicality, and place-identification.
2. Place-prototypicality and place-identification are positively associated.
3. The effects of characteristics of place on collective and intergroup behaviors will be explained by one of the three facets of social identity-based contextualized experience (i.e., self-categorization, place-prototypicality or place-identification).

From a SPACE perspective, group members should demonstrate distinct collective and intergroup behaviors as a function of characteristics of place and the three facets of social-identity based contextualized experience. Moreover, it is expected that viewing place in group-based terms (e.g., perceptions of place-prototypicality) directly shapes sense of belonging (place-identification). Thus, the effects of characteristics of place on collective behavior is expected to be function of perceptions of place-prototypicality (first) and place-identification (second).

A number of characteristics of place likely shape perceptions of the prototypicality of place and sense of belonging, but the present work explores one specific component of local place: level of group representation (i.e., social make-up in terms of numeric group representation). First, consistent with a basic tenets of a SPACE framework, there is evidence that numeric group representation can affect self-categorization [34], perceptions of prototypicality [22], and sense of belonging [14]. Second, there is evidence of a positive association between place-prototypicality and place-identification. Perceived prototypicality of a leader, for example, often partially explains identification with the respective leader [21]. Consistent with past work on prototypicality in the leadership domain [35–37], it would be expected then that there is a positive association between perceptions of prototypicality of place and identification with place. Finally, numeric group representation directly affects collective and intergroup outcomes, such as trust [14] and intergroup integration preferences [38]. Taken together, there are distinct lines of work illustrating that relative level of group representation affects social identity-based experience on the one hand, but also evidence that it affects collective outcomes, on the other hand. Integrating these separate lines of work, the present studies explore the notion that level of group representation within place (i.e., having minority vs. majority-group status) is associated with distinct contextualized experience that is associated with unique collective and intergroup behavioral tendencies.

### Overview of studies

In line with the social identity perspective [31,32] and work rooted in situational cues of context [14,33], the present studies investigate how one component of local place, numeric group representation, affect propensity to view place in group-based terms and also sense of belonging, which (in turn) shape collective and intergroup behavior. First, the present work provides evidence that composition of place or level of group representation shape sense of belonging, which is explained by perceptions of the group-prototypicality of place (Study 1a; Study 5a). In addition, the results illustrate that level of group representation of place affects group members’ comfort and willingness to stay in place via perceptions of group prototypicality of place (place-prototypicality) and sense of belonging (place identification; serial mediation; Studies 1b-1e). The remaining studies were designed to provide evidence for how differing levels of numeric group representation of place (e.g., holding minority vs. majority-group status) is associated with distinct collective and intergroup outcomes as a function of perceptions of group-prototypicality of place and sense of belonging. More specifically, perceptions of the degree of prototypicality of place and sense of belonging help to explain the way group members’ approach (expectations of negative group-based treatment & of procedural justice; Studies 2a-2c), understand (attribution for group-based events; Study 2a & 2b; efficacy of bias-reduction; Study 4a; sense of solidarity; Study 4b), and behave (prejudice; Studies 3a & 3b; collective action; Study 4c) as a function of level of group representation of place. A large number of studies were included to not only demonstrate generalizability across a variety of conditions (i.e., successful replication across differing participant groups, places, and intergroup contexts), but also to provide strong evidence of the diverse set of outcomes shaped by composition of place.

Participants were recruited from Amazon mechanical turk for the equivalent of between $7-$15 per hour (M-turk; Studies 1a-2b 3a, 3b, 4b, & 4c) or from University courses for partial course credit (Studies 2c, 4a, & 5a). Participants for all studies with the exception of Study 5a were pre-selected based on necessary participant demographic characteristics for the respective study (e.g., race/ethnicity; gender) using pre-selection procedures (a screening survey for M-turk or at the beginning semester). None of the participants knew why they were selected for the respective study. All manipulation checks confirmed the expected differences among or between conditions at the *p* < .001 level (see Table 1). Study 3a is the only study in which some participants were dropped as a result of failing the manipulation–check. The City University of New York (CUNY) Integrated Institutional Review Board approved all studies (Human Research Protection Program #34909). All participants were over the age of 18 and the studies contain written or internet-based informed consent—as approved by the IRB. All data were not personally linked to participants. To reduce demand characteristics, some studies included filler items. For ease and clarity of presentation, I have excluded reporting the specifics of these filler items for each of the respective studies. I can send the specifics of these filler items (or the full questionnaire) for any of the studies, upon request. In addition, for tests for indirect effects, Hayes [39,40] procedures were followed, using the PROCESS macro with 5,000 bootstrapped samples (95% bias-corrected intervals). Finally, the recommendations for power of a minimum of 50 participants per cell were followed for all experimental studies [see 41].

**Table 1:** Manipulation-check tests for differences between conditions for all relevant studies.

	Group Representation Condition					
	Low *M (SD)*	High *M (SD)*	*t*	*df*	*Cohen’s d*	95% CI *d*
Study 1a ^a^	1.71 (1.09)	5.96 (1.14)				
Study 1b	2.07 (1.38)	6.20 (1.08)	16.77	99	2.50	[3.64, 4.61]
Study 1c	2.23 (1.47)	6.24 (1.37)	13.90	98	2.82	[3.43, 4.58]
Study 1d	1.44 (1.26)	6.73 (.60)	27.01	109	5.36	[4.91, 5.68]
Study 1e	1.47 (1.29)	6.62 (1.01)	22.13	97	4.46	[4.68, 5.68]
Study 2a	1.58 (1.18)	6.20 (.70)	23.81	98	4.76	[4.23, 5.00]
Study 2b	1.95 (1.54)	6.23 (1.13)	15.48	92	3.16	[3.73, 4.82]
Study 2c	2.01 (1.56)	6.12 (1.24)	15.24	105	2.93	[2.70, 3.23]
Study 3a	2.03 (1.60)	6.17 (.95)	15.11	91	3.15	[3.60, 4.69]
Study 3b Q1^b^	1.90 (1.14)	3.56 (1.34)	6.67	100	1.33	[1.16, 2.15]
Study 3b Q2	4.17 (.65)	6.50 (.88)	15.17	100	3.00	[2.02, 2.63]
Study 4a^c^	3.02 (1.93)	3.72(1.72)				
Study 4b Q1	2.22 (1.68)	6.14 (.92)	13.74	96	2.90	[3.35, 4.48]
Study 4b Q2	2.32 (.95)	4.52 (1.64)	7.81	98	1.64	[1.34, 1.64]
Study 4c Q1	3.63 (1.15)	5.10 (2.42)	3.84	148	0.77	[.49, 1.08]
Study 4c Q2	2.83 (1.54)	4.69 (1.42)	5.90	148	1.25	[1.02, 1.49]

*Note*. *M* = Mean. *SD* = Standard Deviation. Scales ranged from 1–7. All *t* tests were reliable at the *p* < .001 level.

^a^ Study 1a ranged from 1 (*mostly Latino/Hispanic)* to 7 (*mostly White*). Study 1a included three conditions: Low, equal, and high representation, with thus also included an equal representation condition (*M* = 4.01; *SD* = .97) and there was an effect of experimental condition, *F* (2, 147) = 245.30, *η*^2^_*p =*_ .76, and also follow-up comparisons were in the expected direction—with a reliable difference between low and equal representation condition (*p* < .001).

^b^ Study 3b included two manipulation check items. For studies 3b, 5b, and 5c ‘*Q1*’ refers to manipulation check question 1 and ‘*Q2*’ refers to manipulation check question 2.

^c^ Study 4a manipulation check found an effect of experimental condition between the commonality and control condition. Means reflect scores for the control (low) and commonality (high) conditions respectively, *F* (1, 251) = 9.46, *p* = .002, *η*^2^_*p =*_ .03.

## Studies 1a-1e: Level of group representation within place & likelihood of perceiving place in group-based terms

Studies 1a-1e provide an experimental test of whether perceiving divergent levels of group representation within place has a direct effect on perceptions of place-prototypicality and place-identification (sense of belonging). In addition, these studies were designed to explore whether perceptions of the group prototypicality of a place explain the effects of group representation on identification with place. Thus, studies 1a through 1e provide an experimental test of the effect of composition of place on two facets of social identity-based contextualized experience: place-prototypicality and place-identification. Self-identified White (Study 1a & Study 1b), Black (Study 1c), Male (Study 1d) and Female (Study 1e) participants received information about the composition of a place, indicating either low (i.e., minority numerical status) or high (majority numerical status) representation of the respective group membership within place as a part of larger set of information of the respective place. Perceptions of the group-prototypicality of place (place-prototypicality), identification with place (place-identification), and comfort within place were then assessed for the respective group membership. It was hypothesized that group members would report greater place-prototypicality and higher identification with the respective place under conditions of high group-representation, compared to under conditions of equal- or relatively low group-representation within place. In addition, perceptions place-prototypicality was expected to explain the relation between level of group representation within place and identification with place.

### Study 1a

#### Method

One hundred and fifty self-identified White participants (80 women and 70 men; *M* age = 35.91) participated in the study. First, participants were instructed to ‘assume you were in the market for a job’ and that the purpose of the study was to ‘evaluate the attractiveness of an organization.’ Next, all participants were given one page describing a hypothetical organization, called “CCG.” The information included a number of characteristics about the organization, including the culture, benefits, opportunity for high salary, philosophy, and finally commitment to improving employees’ opportunities. Thus, participants were provided with extensive information regarding the organization. A second page provided information about the demographic composition of the organization with respect to race/ethnicity in a pie-chart. The pie-chart served as the composition of place manipulation. The pie chart varied demographic composition of place, such that (for White participants), there was either a low representation (24.90% Whites, 75.10% Hispanic/Latina; i.e., Whites had numerical minority-group status), equivalent representation with another group (50% White, 50% Hispanic/Latino; i.e., control condition), or a high group representation (75.10% White, 24.90% other racial/ethnic groups; i.e., Whites had numerical majority-group status) within space. Although an ‘equal group-representation’ condition is not necessarily ecologically valid, the condition was included as a comparison control-condition to allow for an assessment of the direction of effects for the low and high-group representation conditions. The method and format for the manipulation was adapted from past work investigating representation and social identity [14].

For all conditions, a third page provided some additional information about the culture of the organization. Next, a manipulation-check assessed whether participants viewed the place as being low or high in representation of their group. Specifically, participants were asked ‘What is the racial/ethnic composition of CCG?’, and answered on a 1 (*mostly Latino/Hispanic*) to 7 (*mostly White*) scale.

Using the stem “Thinking in terms of your racial/ethnic group, overall I would say CCG..”, *place-prototypicality* was measured using three items among other filler items (*α* = .94): “…represents what is characteristic about my racial/ethnic group,” “is representative of the unique values of my racial/ethnic group,” and “exemplifies the beliefs that define my racial/ethnic group.” The items were adapted from past work measuring perceptions of prototypicality [28,29]. *Place-identification* was measured using three items (*α* = .91), using the stem “Thinking as a member of your racial/ethnic group about how connected you feel to CCG,”: “…I would feel ‘at home’ at CCG,” “I feel I belong at CCG” and “I identify with CCG.” These items were derived from past work on place identity [6,42]. Participants responded on 1 (*not at all*) to 7 (*very much*) scales for both constructs. Items for both scales were randomized by computer for each individual participant.

#### Results and discussion

A confirmatory factor analysis with oblique rotation was performed to explore whether the place-prototypicality and place-identification items tapped two distinct constructs. Bartlett’s test of sphericity was significant (*X*^2^ = 609.400, *df* = 15, *p* < .01) and the Kaiser-Meyer-Olkin measure was (0.840). Using the Joliffe criteria [43] and a scree test procedure [44; see 45 for guidelines] two factors emerged. Items were considered to load if they had a factor loading of at least .30. The component matrix revealed, as expected, the three place-prototypicality items loaded on one factor (“…characteristic of my group” = .78, “unique values of my group” = .78, & “beliefs that define my group” = .68) and the three place-identification items loaded on the other factor (“. . .at home” = .90, “identify with” = .86, and “I belong” = .85). The factors explained 79.18% of the common variance among the items.

An ANOVA testing for differences in place-prototypicality revealed an effect of representation condition, *F*(2,147) = 46.35, *p* < .001, *η*^2^_*p =*_ .38. Follow-up pairwise comparisons revealed that participants in the high representation condition (*M* = 5.12, *SD* = 1.01) viewed the place as more prototypical of their group, compared to those in the equal (*M* = 4.52, *SD* = .79; *p* < .01) and low representation (*M* = 3.31, *SD* = 1.16; *p* < .001) conditions, respectively. Place-prototypicality was lower in the low representation condition, compared to the equal representation condition, *p* < .001. Similarly, an ANOVA revealed a significant effect of experimental condition on place-identification, *F*(2,147) = 31.62, *p* < .001, *η*^2^_*p =*_ .30. Follow-up pairwise comparisons revealed that participants in the high representation condition (*M* = 5.53, *SD* = .89) reported greater identification with the place, compared to those in the equal (*M* = 4.73, *SD* = 1.15; *p* = .004) and low representation (*M* = 3.58, *SD* = 1.59; *p* < .001) conditions, respectively. Place-identification was lower in the low representation condition, compared to the equal representation condition, *p* < .001.

**Fig 1:**
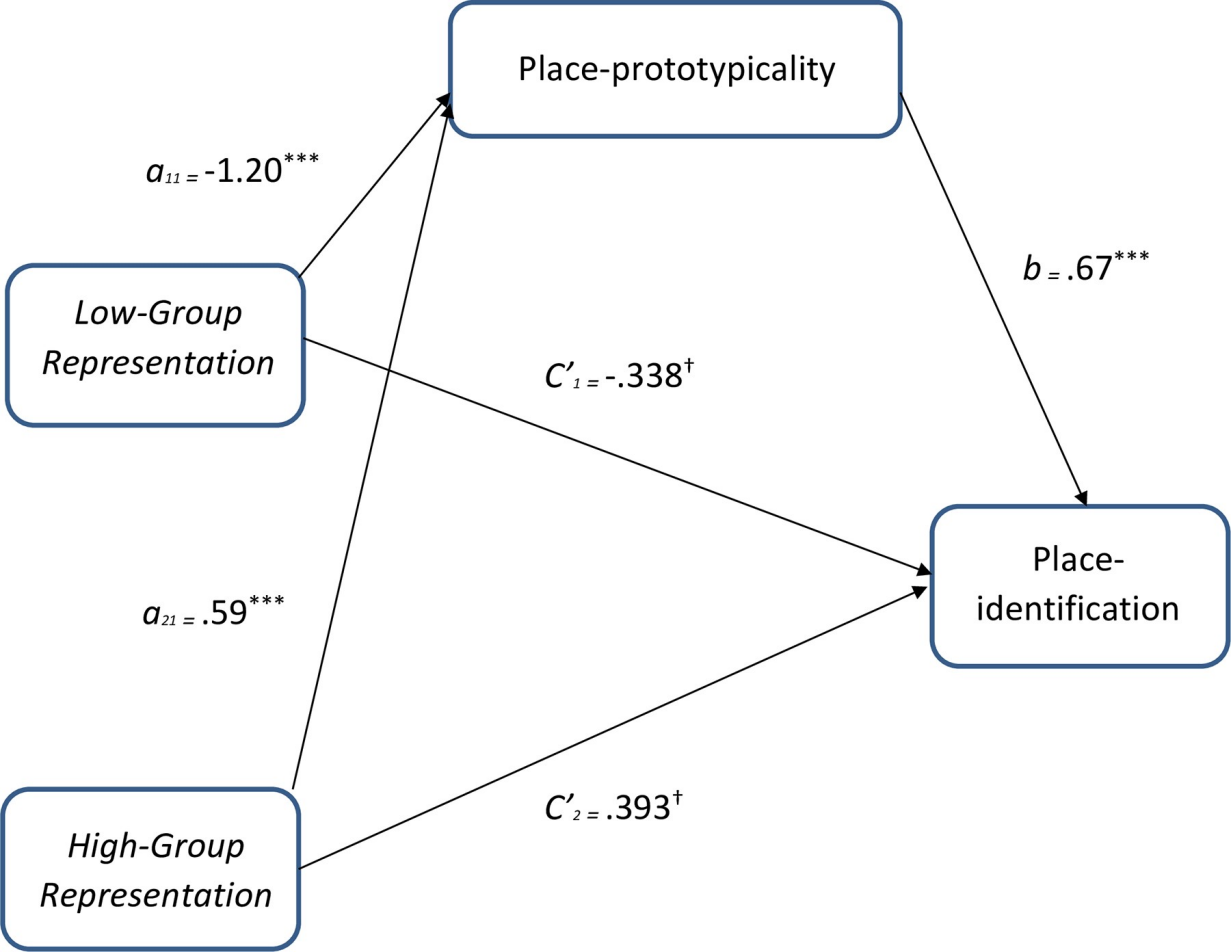
Study 1a mediation analyses for the relative indirect effects of low- and high-group representation conditions (as compared to the control condition—equal representation) on place-identification, via place-prototypicality. ^†^*p* = .07, *p < .05, **p < .01, ***p < .001.

To investigate the hypothesized mediating role of place-prototypicality in explaining the relation between level of group representation within place and sense of belonging (place-identification), a mediation analysis with a multi-categorical independent variable was conducted [46]. As the present work is concerned with relative effects of the experimental conditions (low and high representation), as compared to control (equal representation), a mediation analysis was selected that allows for a test of the relative effects of low and high representation, as compared to control, on place-identification. Thus, following guidelines for analyses with multi-categorical independent variables, this mediation test not only retains all information about how the respective groups differ from one another, but also allows for simultaneous hypothesis testing [46]. To test the significance of the relative indirect effect, a bootstrapping procedure was used and obtained 95% confidence intervals for indirect effects based on 5,000 bootstrap samples. As shown in Fig 1, for the low-group representation condition (as compared to control), there was support for an indirect effect of place-prototypicality on place-identification (*ab*_11_ = -.81, *SE* = .20; 95% CI = -1.26 to -.46). Similarly, for the high-group representation condition (as compared to control), there was support for an indirect effect of place-prototypicality on place-identification (*ab*_12_ = .40, *SE* = .12, 95% CI = .18 to .67). As zero falls outside the interval for both, the indirect effect of level of group representation within place on place-identification via place-prototypicality is significant, demonstrating that increases [decreases] in representation of a group in place directly affect group members’ identification with place, which is explained by variance in perceptions of the group-prototypicality of place (place-prototypicality). Study 1a provides initial evidence that level of group representation has a direct effect on social identity-based contextualized experience. More specifically, the results demonstrate that degree of representation within place shapes identification with place, which is explained by perceptions of place-prototypicality.

Studies 1b-1e were designed to replicate the observed findings, generalize the effects to new target groups, and explore the implications of perceptions of the group-based nature of place (place-prototypicality) and sense of belonging (place-identification) for expectations of comfort and willingness to stay in a respective place. More specifically, White (Study 1b), Black/African-American (Study 1c), Female (Study 1d) and Male (Study 2e) participants received a variety of information about an organization, including composition of place—indicating either low (i.e., minority-group numerical status) or high (i.e., majority-group numerical status) representation of the respective group membership within place—and perceptions of prototypicality, sense of belonging, and perceived comfort of being in the respective place were assessed.

### Studies 1b & 1c

#### Method

One hundred and one White participants (56 men and 45 women; *M* age = 32.13) participated in Study1b and one hundred Black/African-American participants (59 women and 41 men) participated in Study 1c. All procedures and materials were identical to Study 1a, with the exception that the Study 1b group representation manipulation only varied representation of the group as either low (24.90% Whites, 75.10%, other racial/ethnic groups; i.e., minority-group status) or high (75.10% White, 24.90% other racial/ethnic groups; i.e., majority-group status). For Study 1c, the characteristics of the organization indicated there was either a high (75.10% Black, 24.90% other racial/ethnic groups; i.e., majority status) or low level of group-representation for Black/African-American participants (24.90% Blacks, 75.10%, other racial/ethnic groups; i.e., minority status) at the organization.

After the manipulation-check, place-prototypicality (*α* = .94_1b_; *α* = .96_1c_) and place-identification (*α* = .91_1b;_ *α* = .93_1c_) were assessed using measures identical to Study1a. Finally, among several filler items, perceived comfort working at the “CCG” organization was assessed using three items [*α* = .76_1b;_; *α* = .76_1c_; 14]: “I could be myself at a company like CCG”, “My colleagues at CCG would become my close personal friends,” and “I would expect to be treated fairly based on my race/ethnicity.” Participants responded on a 1 (*strongly disagree*) to 7 (*strongly agree*) scale and items for each of the respective scales were randomized for each participant.

#### Studies 1b & 1c results and discussion

**Fig 2:**
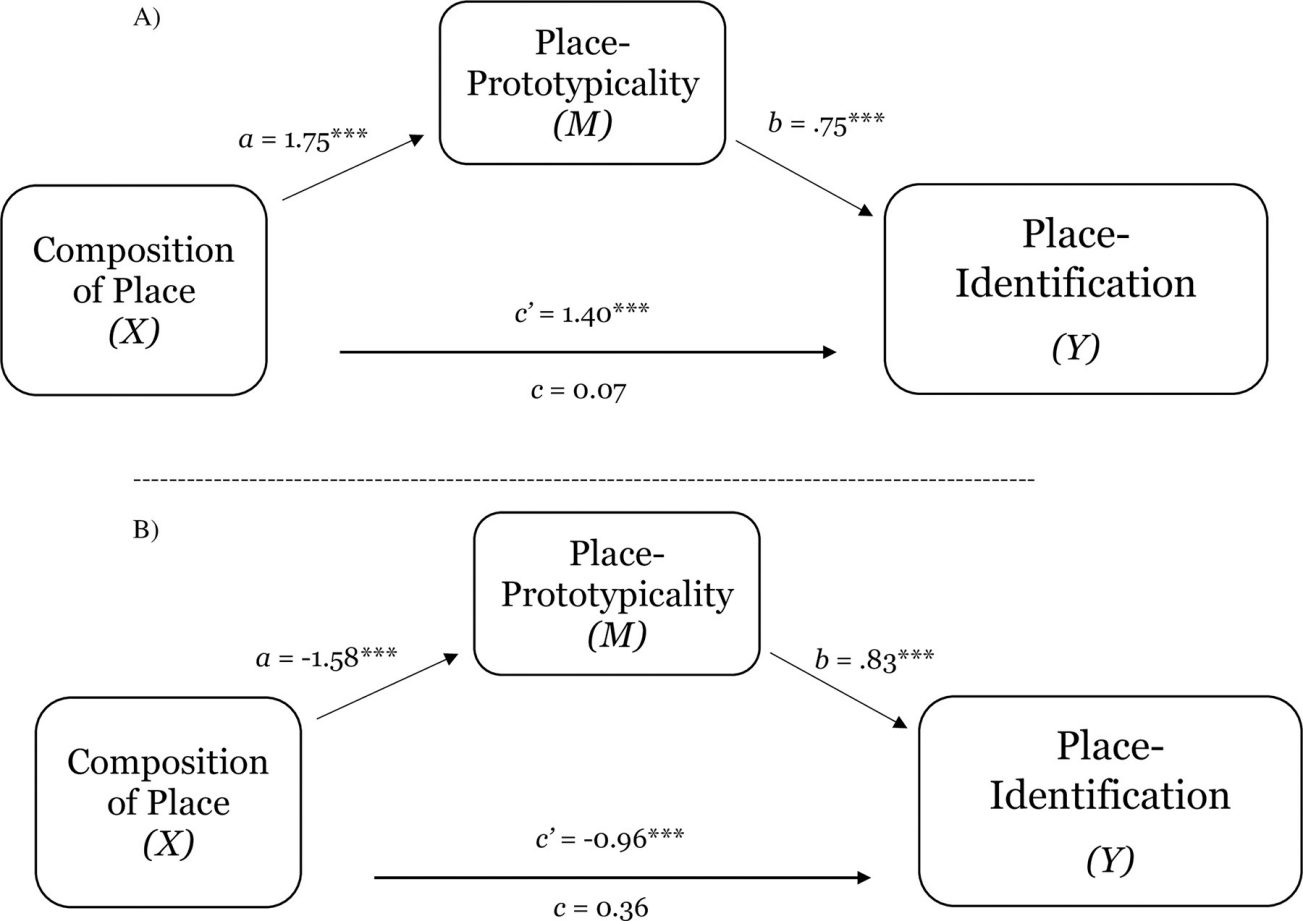
Test of the mediating effect of place-prototypicality on the relation between composition of place and place-identification (sense of belonging) in Study 1b (Panel A) and Study 1c (Panel B). Notes: *p < .05, **p < .01, ***p < .001.

Replicating the results of Study 1a, level of group representation within place had direct effects on perceptions of place-prototypicality, place-identification, and comfort (see Table 2). In addition, as Table 3 shows, composition of place or holding minority or majority-group status had a direct effect on perceptions of place-prototypicality, place-identification (sense of belonging), and comfort. To test the hypothesis that perceptions of group prototypicality of place (place-prototypicality) mediates the relation between level of group representation within place and place-identification, a bootstrapping procedure was used to estimate the indirect effect of composition of place on place-identification through place-prototypicality. The effects of composition of place on place-identification via place-prototypicality was significant for Study 1b and Study 1c. For Study 1b, the point of estimate for the indirect effect was -1.32_1b_ (*SE* = .29) with a 95% bias-corrected confidence interval of -1.95 to -.78 (see Fig 2, Panel A). Similarly, a test of the indirect effect of place-prototypicality on the relation between group representation within place and identification with place was significant (place-prototypicality; estimate for the indirect effect was -1.34_1c_, *SE* = .25; 95% CI = -1.89 to -.89; see Fig 2, Panel B).

**Table 2:** Tests for the effect of group representation on place-prototypicality, place-identification, and outcome variables (Studies 1b-1e).

	Low group representation (Minority status)	High group representation (Majority status)			
	*M (SD)*	*M (SD)*	*t*	*Cohen’s d*	95% CI *d*
Study 1b					
PP	3.39 (1.50)	4.98 (1.34)	5.59***	1.11	[1.02, 2.15]
PI	3.94 (1.79)	4.91 (1.40)	3.02**	0.60	[.33,1.59]
Comfort	4.75 (1.61)	5.36 (1.05)	2.27*	0.44	[.07, 1.13]
Study 1c					
PP	3.46 (1.35)	5.22 (1.47)	6.19***	1.24	[1.19, 2.31]
PI	4.01 (1.53)	5.42 (1.44)	4.72***	0.94	[.81, 1.99]
Comfort	4.43 (1.44)	5.48 (1.19)	3.94***	0.79	[.52, 1.57]
Study 1d					
PP	2.80 (1.34)	5.29 (1.16)	10.48***	1.98	[-2.95, -2.01]
PI	2.68 (1.35)	5.26 (1.33)	10.05***	1.92	[-3.08, -2.06]
Comfort	4.08 (1.55)	5.79 (1.16)	6.67***	1.24	[-2.22, -1.20]
Stay in space	3.94 (1.76)	5.83 (1.25)	6.52***	1.23	[-2.41, -1.29]
Study 1e					
PP	3.30 (1.29)	4.45 (1.42)	4.23***	0.84	[.61, 1.69]
PI	3.51 (1.49)	4.12 (1.31)	2.17*	0.43	[.05, 1.17]
Comfort	4.54 (1.69)	5.34 (1.23)	2.64*	0.54	[.19, 1.40]
Stay in space	4.42 (1.76)	5.16 (1.47)	2.27*	0.45	[.09, 1.38]

*Note*. *M* = Mean. *SD* = Standard Deviation. “PP” refers to place-prototypicality and “PI” refers to place-identification for the respective places. Scales ranged from 1 to 7.

* *p* < .05.

** *p* < .01.

*** *p* < .001. The studies included White (Study 1b), Black/African-American (Study 1c), women (Study 1d) and men (Study 1e) as participants.

**Table 3:** Indirect effects for Study 1b-1e.

	*Point Estimate*	*SE*	*95% CI*
*Study 1b*			
CP->PP ->PI	-1.32	.29	-1.95, -.78*
CP->PP->Comfort	.00	.19	-.35, .43
CP->PI-> Comfort	-.16	.10	-.37, .03
CP->PP->PI->Comfort	.58	.14	.33, .88*
*Study 1c*			
CP->PP->PI	-1.34	.25	-1.89, -.89*
CP->PP->Comfort	.13	.19	-.25, .49
CP->PI->Comfort	.04	.14	-.20, .21
CP->PP->PI->Comfort	.54	.14	.29, .86*
*Study 1d*			
CP->PP->PI	2.00	.23	1.54, 2.53*
CP->PP->Comfort	.49	.31	-.15, 1.10
CP->PI->Comfort	.24	.14	.02, .58*
CP->PP->PI->Comfort	.86	.26	.41, 1.45*
CP->PP->Stay in space intentions	.33	.35	-.39, 1.01
CP->PI->Stay in space intentions	.31	.18	.03, .74*
CP->PP->PI->Stay in space intentions	1.10	.32	.55, 1.81*
*Study 1e*			
CP->PP->PI	-.83	.21	-1.31, -.44*
CP->PP->Comfort	-.24	.20	-.67, .13
CP->PI->Comfort	-.16	.15	-.48, .12
CP->PP->PI->Comfort	.55	.19	.23, .97*
CP->PP->Stay in space intentions	.15	.12	-.08, .40
CP->PI->Stay in space intentions	-.03	.04	-.14, .03
CP->PP->PI->Stay in space intentions	.13	.09	-.04, .35

*Note*. *CP* = Composition of place. *PP* = Place-prototypicality. PI = Place-identification. CI = confidence interval.

*Indirect effect is reliable at *p* < .05 (excluding zero).

A critical hypothesis of the present work is that the characteristics of a given place directly shape group-based perceptions of the respective place, which (in turn) is associated with sense of belonging. Sense of belonging, then, directly affects collective and intergroup behavior.

**Fig 3:**
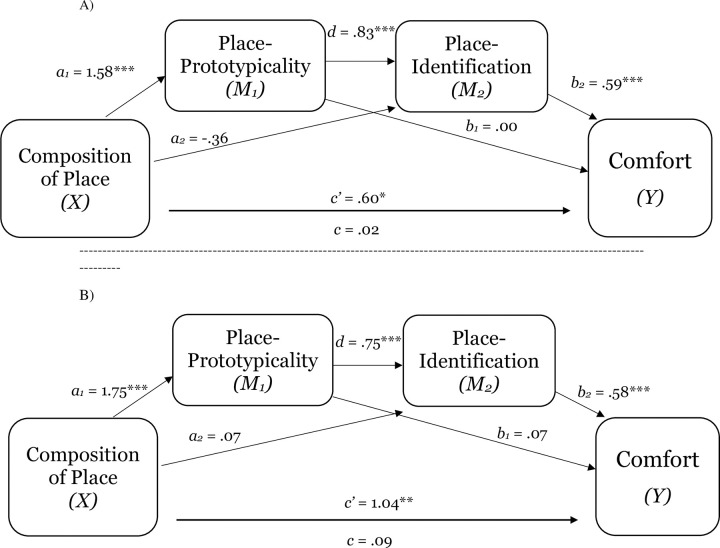
The serial mediating effect of place-prototypicality and place-identification in the relation between composition of place and comfort in Study 1b (Panel A) and Study 1c (Panel B). Notes: *p < .05, **p < .01, ***p < .001; All presented effects are unstandardized; c’ is direct effect of composition of place on comfort; c is total effect of composition of place; *d* is effect of place-prototypicality on place-identification (sense of belonging).

To test the hypothesis that perceptions of group prototypicality of space (place-prototypicality) and sense of belonging (place-identification) mediate the relation composition of place and comfort, a bootstrapping procedure was used to estimate the indirect effect of composition of place on comfort through place-prototypicality and place-identification (sequential mediation). For Study 1b, composition of place had a reliable effect on comfort (total effect; *B* = .60, *p* < .001). When the mediators were included in the analysis this coefficient was not reliable (direct effect, *B* = 0.02, *p* = .91). Moreover, the point estimate of the indirect effect of composition of place on comfort via a progression of place-prototypicality and then place-identification was 0.79 (*SE* = 0.21) with 95% bias-corrected confidence interval of 0.42 to 1.27 (see Fig 3; Panel A). These results were replicated in Study 1c, the point estimate of the indirect effect of composition of place on comfort via a progression of perceptions of place-prototypicality and then place-identification was 0.54 (*SE* = .14) with 95% bias-corrected confidence interval of .29 to .86 (see Fig 3; Panel B).

Thus, across two different racial/ethnic groups, the results provide clear and similar pattern of effects regarding the effects of level of group representation on social identity-based contextualized experience in the form of place-prototypicality and place-identification. Studies 1d and 1e were designed to replicate the observed findings within a new intergroup context (gender) and space (restaurant), as well as add a new outcome measure (intentions to stay in place).

### Studies 1d & 1e

#### Method

Studies 1d and 1e were designed to examine the effects of group representation for two additional target groups: women (Study 1d) and men (Study 1e). One hundred twelve self-identified women (*M* age = 37.85; 71% self-identified as White, 15% Black/African-American, 6% Latina/Hispanic, 5% Asian, and 3% bi-racial/multi-racial) participated in Study 1d and one hundred self-identified men (*M* age = 37.62; 77% self-identified as White, 10% Asian, 8% Black/African-American, 4% Latino/Hispanic, and 1% bi-racial/multi-racial) participated in Study 1e.

All participants were given one page describing a hypothetical restaurant called “Sam’s diner,” and told to assume that they were looking for a place to eat. Based on the menu, as well as online reviews, the restaurant seemed “very, very good!” An additional paragraph described general practices of the restaurant, including reservation requirements (“walk-ins encouraged”), seating capacity (“50 people”), and commitment to extraordinary customer service. On the next page, participants were told to assume they decided to go to the restaurant for dinner and when they entered the restaurant, it was almost completely full. More specifically, participants were informed that “When you arrive to the restaurant, it is almost completely full (though the host tells you there are seats available for you).” The next page informed participants that the restaurant (including all staff and people eating) had a specific gender composition. More specifically, the page said “As you look around the restaurant, you notice it is mostly filled with [males/females].” For Study 1d, a graph depicted composition of place varied representation of the group, such that there was either a low (10% female, 90%, male; i.e., numerical minority-group status) or high (90% female, 10% male; i.e., numerical majority-group status) number of women in the respective place. For Study 1e, the graph indicated there was a high (90% males, 10% females; i.e., majority status) or a low (10% males, 90% females; minority status) representation of men in the place.

On the next page, a manipulation-check assessed whether participants viewed the place as being low or high in representation of their group. Specifically, participants were asked ‘What is the gender composition of the restaurant?’, and answered on a 1 (*mostly male*) to 7 (*mostly female*) scale for Study 1d and on a 1 (*mostly female*) to 7 (*mostly male*) scale for Study 1e. On the following page, place-prototypicality (*α* = .94_1d;_ 96_1e_) and place-identification (*α* = .96_1d;_ 90_1e_) were measured using items identical to study 1b (adapted to gender). Comfort was assessed using three items (*α* = .88_1d;_ 90_1e_): “I could be myself at this restaurant”, “I could relax and ‘be me’ at this restaurant,” and “I would feel comfortable being myself at this restaurant.” Finally, one item assessed participants’ intentions to stay in place: “I would decide to stay and eat at this restaurant” Both comfort and decision to stay in space were assessed with participants responding on a 1 (*strongly disagree*) to 7 (*strongly agree*) scale.

#### Studies 1d & 1e results and discussion

**Fig 4:**
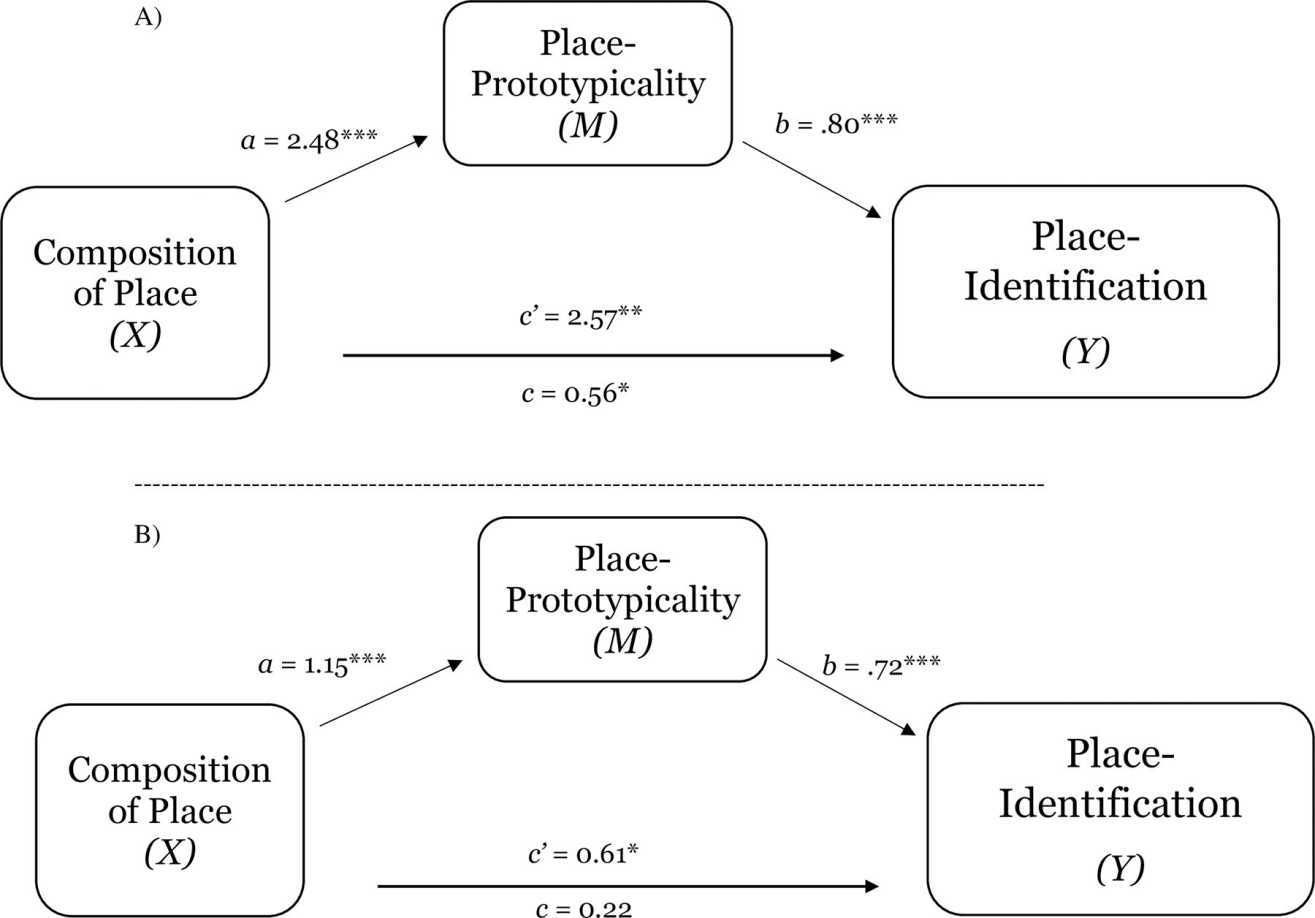
Test of the mediating effect of place-prototypicality on the relation between composition of place and place-identification (sense of belonging) in Study 1d (Panel A) and Study 1e (Panel B). Notes: *p < .05, **p < .01, ***p < .001.

**Fig 5:**
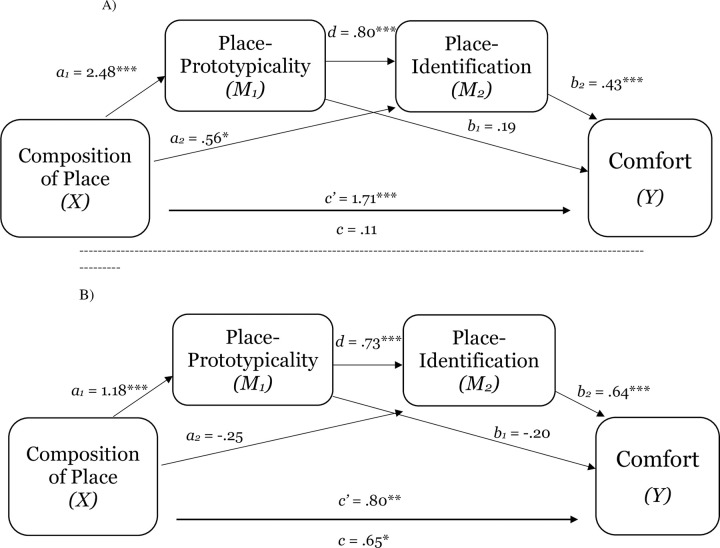
The serial mediating effect of place-prototypicality and place-identification in the relation between composition of place and comfort in Study 1d (Panel A) and Study 1e (Panel B). Notes: *p < .05, **p < .01, ***p < .001; All presented effects are unstandardized; c’ is direct effect of composition of place on comfort; c is total effect of composition of place; *d* is effect of place-prototypicality on place-identification (sense of belonging).

**Fig 6:**
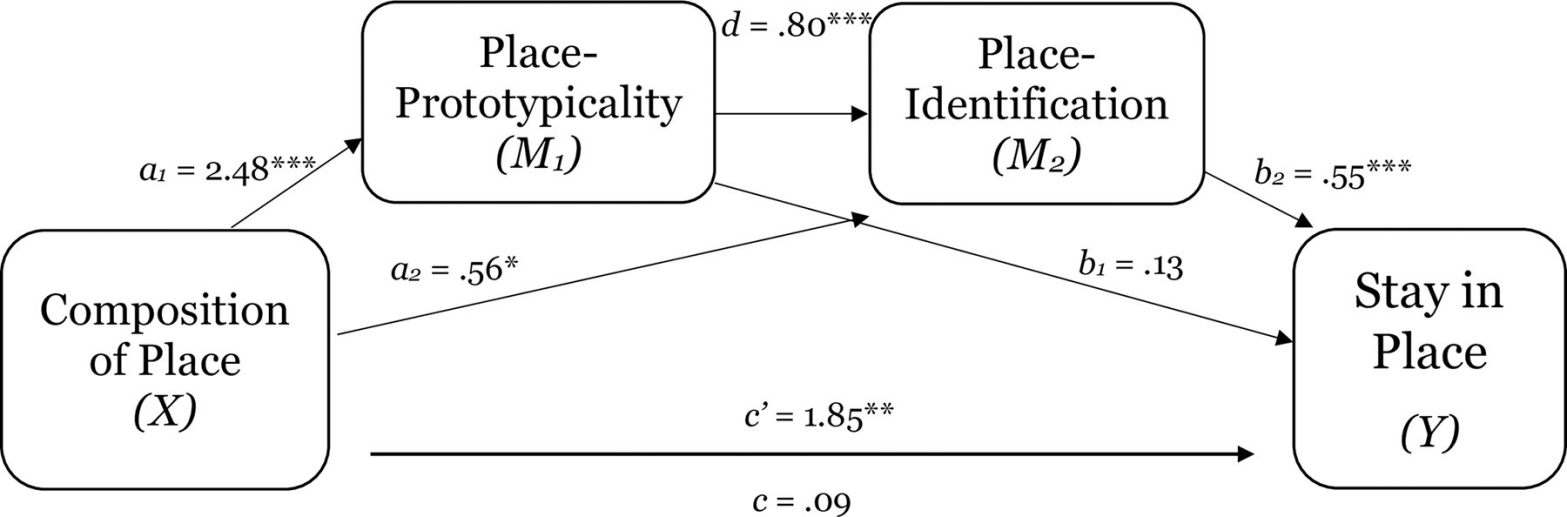
The serial mediating effect of place-prototypicality and place-identification in the relation between composition of place and intentions to stay in place in Study 1d. Notes: *p < .05, **p < .01, ***p < .001; All presented effects are unstandardized; c’ is direct effect of composition of place on comfort; c is total effect of composition of place; *d* is effect of place-prototypicality on place-identification (sense of belonging).

Providing further evidence of the implications of level of group representation of place for perceptions of place-prototypicality, place-identification, and comfort, Studies 1d and 1e replicated the findings of Studies 1a-1c (see Table 2). For both Study 1d and 1e, a test of the indirect effect of perceptions of group prototypicality of place on the relation between group representation within place and place-identification was significant (place-prototypicality; estimate for the indirect effect was 2.00_1d_, *SE* = .23; 95% CI = 1.54 to 2.53; see Fig 4, Panel A; estimate -.83_1e_, *SE* = .21; 95% CI = -1.31 to -.44; see Fig 4, Panel B). Also replicating the results from Studies 1a and 1b, across both studies, the results demonstrate that place-prototypicality and place-identification (sequential mediation) explained the effect of representation within place on comfort (estimate .86_1d_, *SE* .26; 95% CI = .41 to 1.45; see Fig 5, Panel A; estimate .55_1e_, *SE* .19; 95% CI = .23 to .97; see Fig 5, Panel B). Consistent with the notion that perceptions group-based nature of place shape sense of belonging, which (in turn) shape collective outcomes, Study 1d also found that place-prototypicality and place-identification (sequential mediation) explained the effect of representation within place on intentions to stay in place (estimate 1.10, *SE* .32; 95% CI = .55 to 1.81; see Fig 6).

To obtain a better estimate of the true effect size of composition of place on the proposed outcomes, a mini-meta-analysis was conducted [47; www.mini-metaanalysis.com] for Studies 1b, 1c, 1d, and 1e. The averaged corrected standardized mean difference for the effect of composition of place on the outcomes was as follows: on place-prototypicality was *d* = 1.26. 95% CI = [1.04, 1.47], *Z* = 11.56, *p* < .001, on place-identification was *d* = .91. 95% CI = [.71, 1.12], *Z* = 8.69, *p* < .001, and on comfort was .74. 95% CI = [.54, .94], *Z* = 7.27, *p* < .001. Finally, the indirect effect of composition of place on place-identification via place-prototypicality was *b* = -1.33, 95% CI = [-1.57,-1.09], *Z* = -10.98, *p* < .001.

Taken together, across two intergroup contexts (i.e., race/ethnicity and gender) and settings (organization and restaurant), the results of Studies 1a through 1e provide experimental support for the role of degree of group representation in shaping propensity to view places in group-based terms, in particular perceptions of place-prototypicality. Moreover, the studies illustrate that the extent to which a group member views a place (or the characteristics of a place) as being prototypical of their group directly affects the person’s sense of belonging and (in turn) collective outcomes (e.g., comfort and intentions to stay in the respective place). That is, consistent with a group-based approach to place, the findings illustrate that perceptions of prototypicality serve as an antecedent to sense of belonging and group-based behavioral tendencies.

More generally, the findings further support a context-driven account of collective behavior, which would suggest distinct and dissimilar patterns of behavior as a function of characteristics of place and social-identity based contextualized experience. Collective preferences were a function of level of group representation within place, and not group membership, *per se*. Men and Whites (majority groups in that national context) with minority-group status in the local context showed the same pattern of collective preferences as women and Black/African-Americans. Thus, expecting or having minority vs. majority-group status—as a function of composition of place—is associated with distinct group-based perceptions and perceived sense of belonging in the respective place. Therefore, the findings provide evidence for distinct psychological orientations as a function of group-based status within local context—rooted in group-based perceptions of place and sense of belonging. The remaining studies were designed to explore how level of group representation or composition of place in a local context shape a wide range of collective and intergroup outcomes, encompassing how a group member approaches, understands, and behaves (tendencies). As a start, Studies 2a-2c examine how composition of place affects expectations for group-based treatment and interpretation of group-based events.

## Studies 2a-2c: Level of group representation within place, expectations of group-based treatment, and attribution for group-based events

Studies 2a-2c explore how diverging levels of representation affects expectations of negative group-based treatment and propensity to make group-based attributions (Studies 2a & 2b), as well as minority-group expectations of procedural justice and willingness to partner with law enforcement organizations (Study 2c). Studies 2a-2c also extend the present work by testing how composition of place affects the third facet of social-identity based contextualized experience: the way one conceives of oneself (self-categorization) and, as such, the lens in which a person interprets group-based events.

The social identity perspective suggests that people can define themselves at differing levels of abstraction, ranging, for example, from a subordinate level as a unique individual (i.e., personal identity; “I’ vs. “you”) to an intermediate level as a member of a group [i.e., social identity; “We” vs. “They”; 31,32]. Self-categorization is rooted in context and to the extent that the others present in a context varies (changing with whom a person can compare), it would be expected that the way a person conceives of themselves in these respective contexts would also differ [i.e., category salience varies; 32].

One implication of viewing the world through the lens of one’s group membership is a person may be especially likely to make group-based attributions. There are two types of perception bias with respect to discrimination [48]: a tendency to see less discrimination (minimization bias) and a tendency to see more discrimination (vigilance bias). Anticipating differing levels of group representation within place was expected to have a direct effect on expectations of group-based treatment and attribution, which would be explained by shifts in self-categorization. More specifically, it was hypothesized that degree of group representation within place should directly affect self-categorization, such that under conditions of low group-representation, participants should be more likely to view themselves in terms of their social identity (i.e., self-categorization at the level of social identity), compared to under conditions of high group-representation. In addition, to the extent that a person views themselves in terms of social identity, it would be expected that they should be more likely to interpret the world at the level of social identity (i.e., through the lens of the respective group membership) and as such be more likely to make group-based attributions during group-relevant events (i.e., vigilance bias).

### Studies 2a & 2b

#### Method

Ninety-eight self-identified White (*M* age = 38.56; 50% men and 50% women) participants participated in Study 2a and Ninety-four self-identified Black/African-American participants (*M* age = 32.27; 62% women and 38% men) participated in Study 2b. Participants were instructed to “imagine they had been accepted to a University” and told the purpose of the study was to understand how people “respond to everyday events on campus.” They were then given a one page describing background information about the University. The page included a variety of characteristics of the University, including the student-faculty ratio, commitment to small classrooms, building a close-knit community, and providing students with hands-on experience in the lab and in the field. The next page provided information about the demographic composition of the entire University with respect to race/ethnicity. For Study 2a, a pie chart varied representation of the group within place, such that there was either a low representation of Whites (30.70% other racial/ethnic groups, 69.30% Blacks/African-American; i.e., Whites had numerical minority-group status) or a high representation of Whites (69.30% Whites; 30.70% other racial/ethnic groups; i.e., Whites had numerical majority-group status). For Study 2b, the manipulation indicated there was either a low representation of Blacks (30.70% other racial/ethnic groups; 69.30% Whites; minority-group status) or a high representation of Blacks (69.30% Black, 30.70% other racial/ethnic groups; i.e., majority-group status) in the respective place.

On the next page, a manipulation-check assessed whether participants viewed the place as being low or high in representation of their group. Specifically, participants were asked ‘What is the racial/ethnic composition of the University?’, and answered on a 1 (*mostly non-White*) to 7 (*mostly White*) scale for Study 2a or on a 1 (*not many Black/African-American students*) to 7 (*many Black/African-American students*) scale for Study 2b.

On the following page, participants were instructed to consider living on campus, walking around the University, and interacting with other students. Self-categorization at the level of social identity was assessed using three items adapted from past work [49]: “To what extent would you think about your own racial/ethnic group identity?” (1 = *not at all*; 7, *very much*), “I would see myself in terms of my racial/ethnic group identity” (1 = *never*; 7, *very frequently*), and “I would probably be more likely to than usual to think about myself in terms of my racial/ethnic identity” (1 = *strongly disagree*; 7, *strongly agree*; *α* = .80_2a_; 85_2b_). Next, to assess expectations of group-based treatment, participants were asked how concerned they would be about others treating them negatively based on their racial/ethnic group membership. Using the stem, “Because of my racial/ethnic group membership, I would be concerned about…”, four items assessed expectations for negative group-based treatment [*α* = .95; 50]: “…being ignored, overlooked, or not given service,” “…being treated rudely or disrespectfully,” “…feeling excluded from social events,” and “…being insulted, called a name or harassed.” Participants responded on a 1 (*not at all concerned*) to 7 (*very concerned*) scale (*α* = .81_2a;_ 90_2b_).

To assess propensity to make group-based attributions, participants were asked to make a judgement regarding perceived discrimination based on racial/ethnic group membership. Specifically, on the next page, participants imagined the following situation [derived from past work; 51,52]:

Suppose that it’s the beginning of the semester and you need an “add code’ for a course required for your major. You stop by the professor’s office and politely ask to be let into the class. To your disappointment, the professor turns you down and says, ‘Sorry, but I just can’t give you an add code.’ Later that day, you talk to a friend and he/she is surprised the professor didn’t let you into the class because she heard (but was not 100% sure) that the professor let another student in after you met with the professor.

Perceived group-discrimination was measured using three items [*α* = .86_2a;_ 92_2b_; 51]: “To what extent was the professor’s behavior due to racial/ethnic discrimination?” (1 = *not at all*; 7 = *very much*), “The professor’s behavior was biased against my racial/ethnic group” (1 = *not at all likely*; 7 = *very likely*), and “The professor’s actions regarding the ‘add code’ were due to racial/ethnic discrimination” (1 = *not at all likely*; 7 = *very likely*). Finally, two items assessed the extent to which participants viewed the professor’s actions as a result of external factors (i.e., situational attribution; *r* = .66_2a_; *r* = .62_2b_): “The professor’s decision was about the situation” (1 = *strongly disagree*; 7 = *strongly agree*), and “What was the relative importance of situational factors in determining the professor’s decision?” (1 = *extremely unimportant*; 7 = *extremely important*).

#### Studies 2a & 2b results and discussion

**Fig 7:**
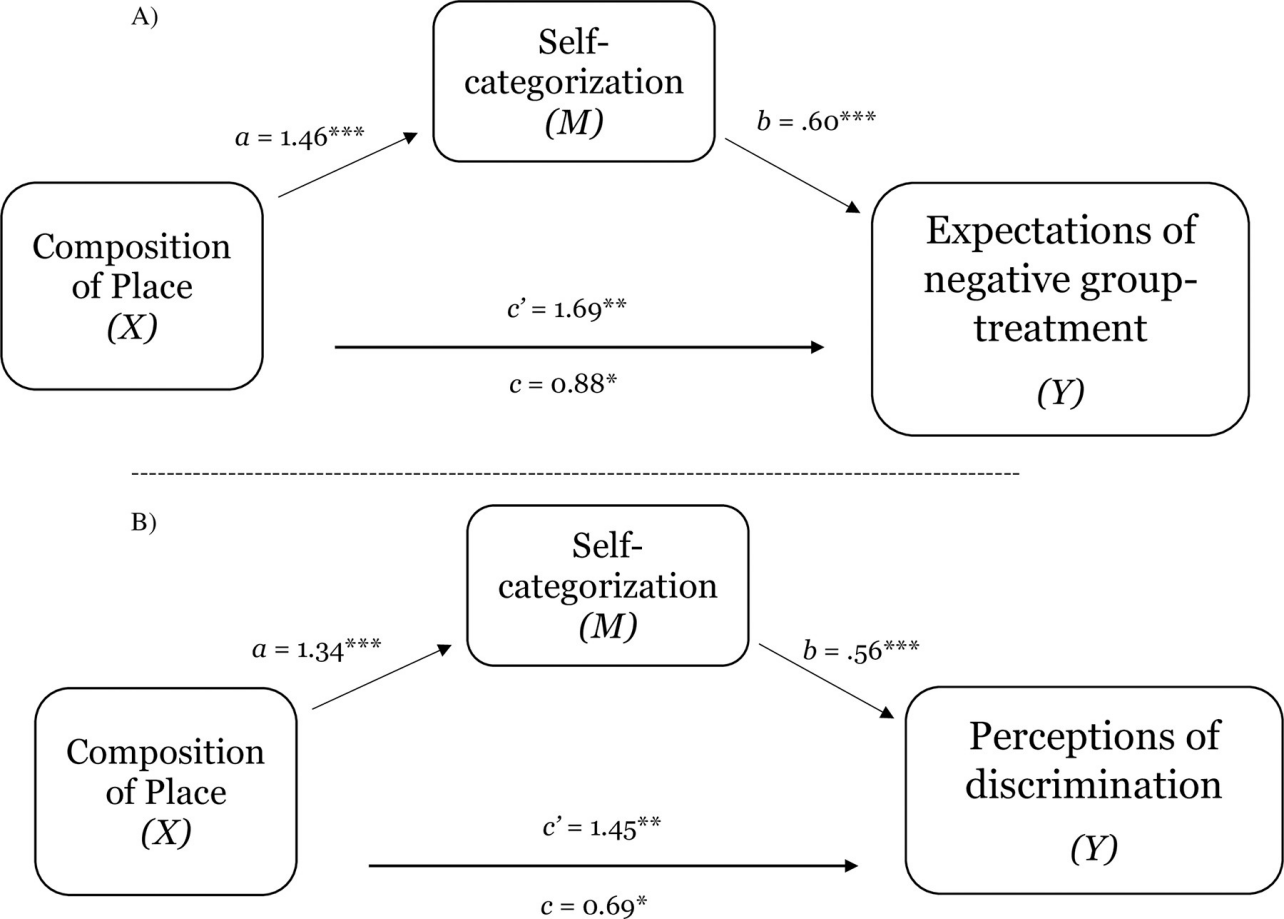
Test of the mediating effect of self-categorization on the relation between composition of place and expectations of negative group-based treatment (Panel A) and perceptions of discrimination (Panel B) in Study 2a. Notes: *p < .05, **p < .01, ***p < .001.

**Fig 8:**
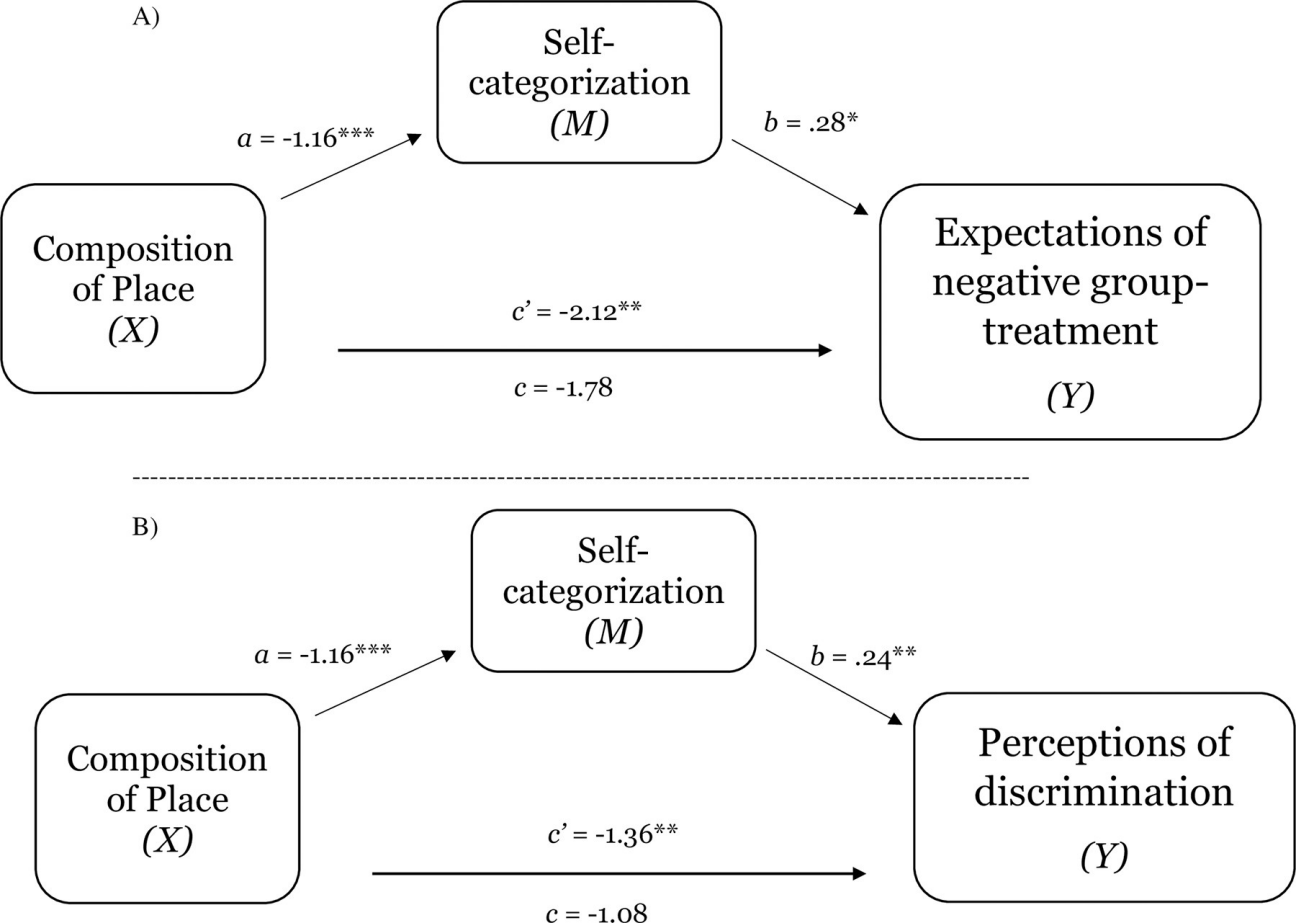
Test of the mediating effect of self-categorization on the relation between composition of place and expectations of negative group-based treatment (Panel A) and perceptions of discrimination (Panel B) in Study 2b. (Panel A). Notes: *p < .05, **p < .01, ***p < .001.

As Table 4 demonstrates, across both Study 2a and 2b, composition of place had a direct effect on self-categorization, expectations of negative group-based treatment, and perceived group discrimination, but not situational attribution. Across both Study 2a and Study 2b, a test to explore whether self-categorization explained the effects of levels of group representation within place on expectations of negative group-based treatment was significant (estimate .81_2a_, *SE* = .23; 95% CI = .45 to 1.30; estimate -.33_2b_, *SE* = .17; 95% CI = -.77 to -.06; see Figs 7 and 8). Similarly, results demonstrated that self-categorization explained the effect of group representation within place on perceptions of discrimination (estimate .76_2a_, *SE* .19; 95% CI = .42 to 1.24; estimate -.27_2b_, *SE* .18; 95% CI = -.72 to -.01; see Figs 7 and 8). Across two different racial/ethnic groups, the findings of Studies 2a and 2b demonstrate that level of group representation within place has a direct effect on not only expectations of group-based treatment, but also attributions (understanding) of group-based events. Thus, these studies provide initial evidence that, even before entering a place, composition of place can set a foundation for the way a group member approaches the respective place by shaping expectations of how they will be treated based on their group membership.

**Table 4:** Tests for the effect of group representation on self-categorization and outcome variables (Studies 2a, 2b, & 2c).

	Low group representation (Minority status)	High group representation (Majority status)			
	*M (SD)*	*M (SD)*	*t*	95%CI	*Cohen’s d*
Study 2a					
SC	4.52 (1.55)	3.18 (1.35)	4.58***	0.76, 1.93	0.92
Expec. Neg treat.	3.57 (1.88)	1.87 (1.16)	5.32**	1.06, 2.32	1.08
Perc. Grp. Disc.	3.82 (1.87)	2.36 (1.56)	4.19***	0.76, 2.15	0.84
Sit. Attrib.	4.22 (1.48)	4.56 (1.30)	1.17		
Study 3b					
SC	5.29 (1.13)	4.13 (1.47)	4.31***	0.62, 1.69	0.88
Expec. Neg treat.	4.75 (1.22)	2.63 (1.71)	7.00***	1.52, 2.72	1.42
Perc. Grp. Disc.	4.82 (1.12)	3.46 (1.61)	4.82***	0.80, 1.92	0.98
Sit. Attrib.	4.01 (1.27)	4.36 (1.35)	1.39		
Study 3c					
PP	3.16 (1.22)	3.84 (1.34)	2.72**	.29, .77	0.53
PI	3.26 (1.54)	4.11 (1.90)	2.53*	.30, .90	0.49
Expect of Proc.just.	4.31 (1.72)	5.36 (1.49)	3.37**	.35, .96	0.65
Partner with Police	4.68 (1.27)	5.27 (1.51)	2.19*	.16, .68	0.42

*Note*. *M* = Mean. *SD* = Standard Deviation. “SC” refers to self-categorization at the level of social identity, “Expec. Neg. treat.” refers to expectations of negative group-based treatment, and “Perc. Grp. Disc. refers to perceived group discrimination. “Expect of Proc Just” refers to expectations of procedural justice and “SI” refers to space-identification for the respective spaces. Scales ranged from 1 to 7.

* *p* < .05.

** *p* < .01.

*** *p* < .001. Studies included White (Study 2a), Black/African-American (Study 3b), and Latinx (Study 3c) participants.

Beyond shaping expectations, composition of place also influences attributions made within the respective place, such that differing levels of group representation are associated with distinct patterns of propensity to make group-based attributions. The literature on perceptions of discrimination has moved toward a framework that seeks to identify the personal, situational, and structural factors that shape individuals’ likelihood of viewing themselves as victims of discrimination [48]. A socio-structural framework suggests that situational cues and societal group status (i.e., minority vs. majority) can shape perceptions of bias [48]. The current findings contribute and compliment this work by suggesting that self-categorization may also help to explain why group members make group-based discriminatory attributions. Beyond expectations of negative group-based treatment, level of group representation of place was also hypothesized to affect expectations of fair treatment (i.e., procedural justice) and willingness to partner or work with law enforcement or authority organizations.

### Study 2c

#### Method

One hundred and seven self-identified Latinx participants (i.e., Hispanic/Latina/Latino; 63% women and 37% men; *M* age = 22.23) participated to fulfill one option of an introductory psychology course. Participants were informed that the researchers were interested in “impressions about police departments.” In all conditions, participants were then given one page of information focused on the ‘mission of the police department’, which included text on the department’s commitment to enhancing quality of life, partnering with the community, preserving peace, reducing fear, maintaining order, as well as treating every citizen with courtesy, professionalism, and respect. A second page provided information about the general demographic make-up of the police department. The composition of place manipulation varied representation of the group, such that there was either a low representation of Latinx (24.90% Hispanic/Latina, 75.10% White; i.e., minority-group status) or a high representation of Latinx (75.10% Hispanic/Latina, 24.90% Whites; i.e., majority-group status). On the next page, a manipulation-check assessed whether participants viewed the police department as being low or high in representation of their group. Specifically, participants were asked ‘What is the racial/ethnic composition of the police department?’, and answered on a 1 (*mostly Latina/o/Hispanic*) to 7 (*mostly White*) scale. On the following page, place-prototypicality (*α* = .90) and place-identification (*α* = .91) were measured using items identical to previous studies. Next, participants were asked to “consider how you would be treated by the police department” and four items assessed expectations of procedural justice. Using the ‘I would expect’ stem, the four items were as follows: “…the procedures used by this police department in dealing with my racial/ethnic group to be fair,” “…the policies of this police department to be just,” “…this police department to develop policies that treat my racial/ethnic group in an unbiased way.” Participants responded on a 1 (*strongly disagree*) to 7 (*strongly agree*) scale (*α* = .93). In addition, willingness to partner with the police department was measured using five items. More specifically, participants were asked to consider the police department they read about earlier and asked, ‘if the situation arose, how likely would you be to…”: “…call the police to report a crime that was occurring,” “report suspicious activity by the police,” “..attend meetings with police to build community-police partnerships,” “…talk to an individual police officer on the street,” and “…cooperate with the police department.” Participants responded on a 1 (*not at all likely*) to 7 (*extremely likely*) scale (*α* = .84). The items and response format were adapted from past work exploring procedural justice and willingness to partner with law enforcement [53].

#### Results and discussion

**Fig 9:**
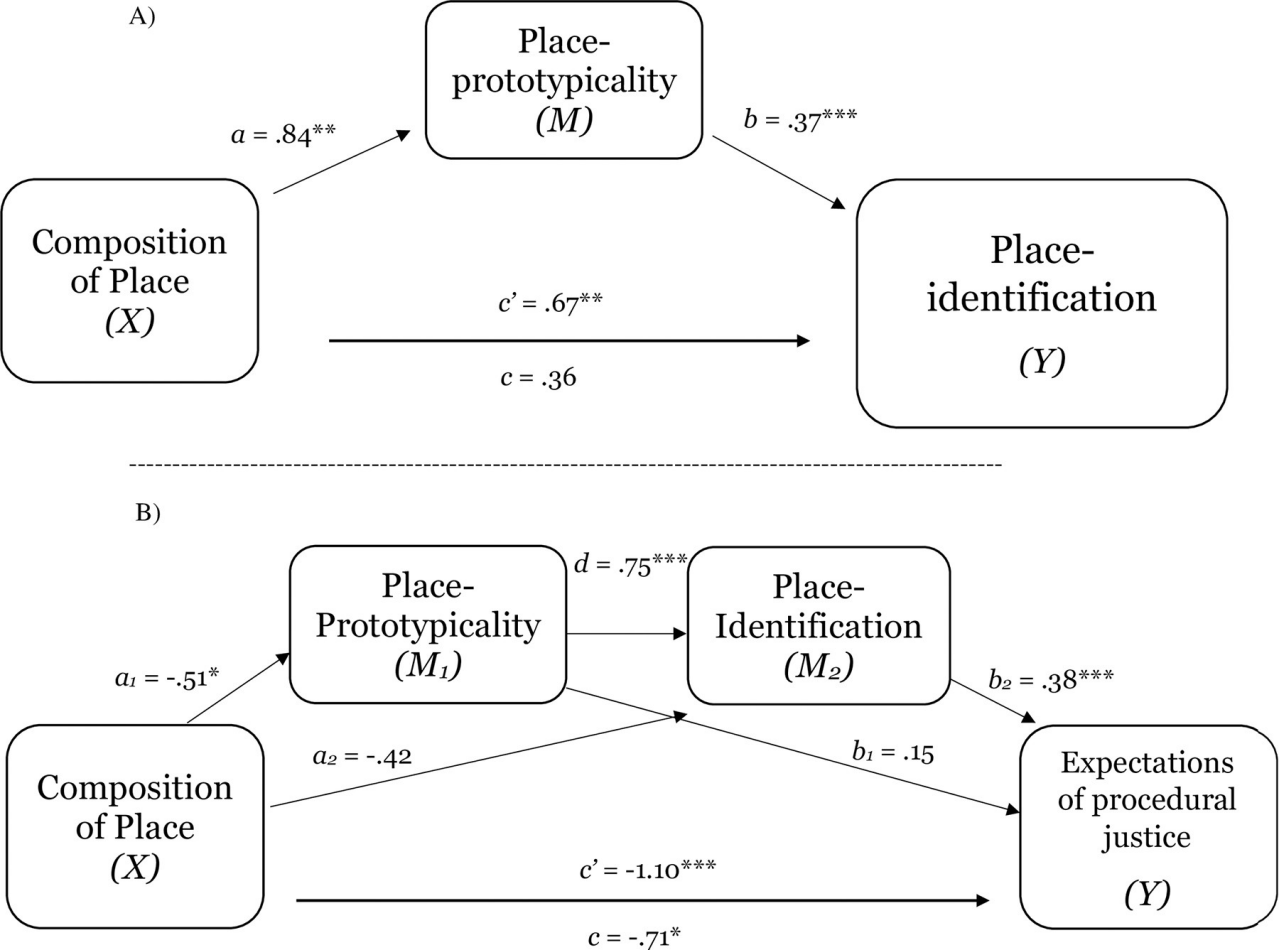
Test of the mediating effect of place-prototypicality on the relation between composition of place and place-identification in study 2c (Panel A). Test of the serial mediating effect of place-prototypicality and place-identification in the relation between composition of place and expectations of procedural justice in study 2c (Panel B). Notes: *p < .05, **p < .01, ***p < .001.

**Fig 10:**
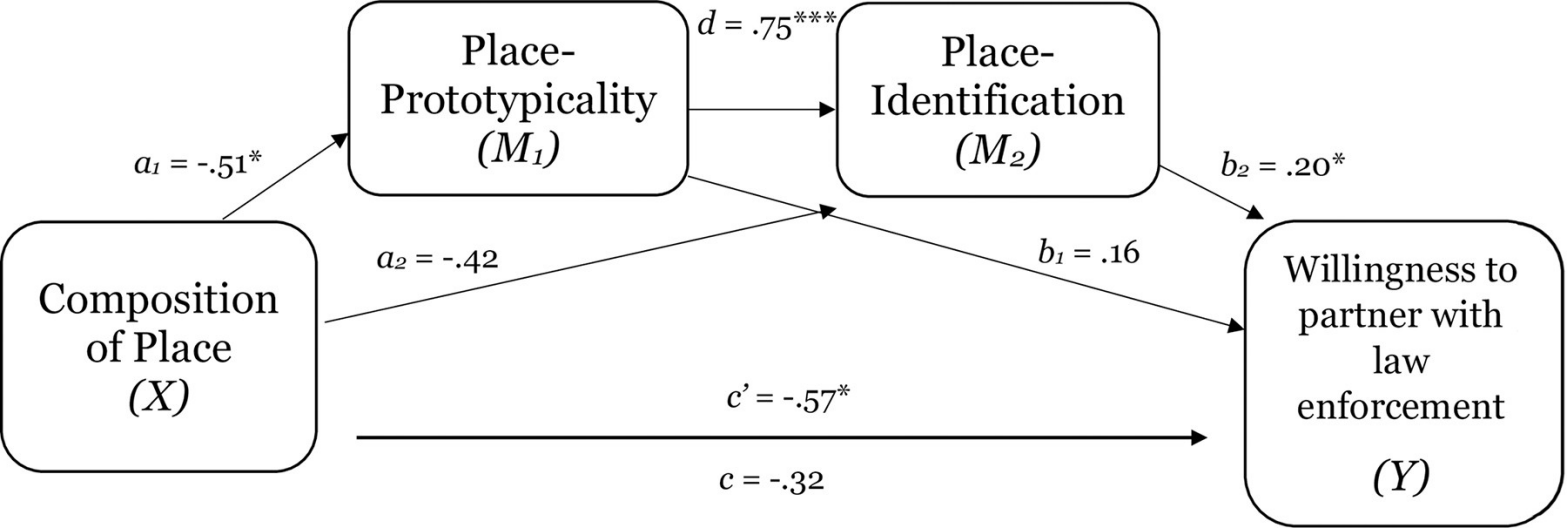
Test of the serial mediating effect of place-prototypicality and place-identification in the relation between composition of place and willingness to partner with law enforcement in study 2c. Notes: *p < .05, **p < .01, ***p < .001.

As Table 4 demonstrates, composition of place had a direct effect on perceptions of place-prototypicality, place-identification, expectations of procedural justice, and willingness to partner with law enforcement. In addition, replicating the findings of the previous studies, perceptions of the group-prototypicality of place explained the effect of composition of place on sense of belonging (see Table 5 and Fig 9). Moreover, consistent with a group-based approach to place and replicating the results of previous studies, the results demonstrate that perceptions of place-prototypicality and place-identification (sequential mediation) explained the effect of level of representation of place on expectations of procedural justice (estimate -.08, SE .05; 95% CI = -.20 to -.01; see Table 5 and Fig 9) and willingness to partner with law enforcement (estimate—.08, SE .07; 95% CI = -.19 to -.01; see Table 5 and Fig 10).

**Table 5:** Indirect effects for Studies 2a-5c.

	*Point Estimate*	*SE*	*95% CI*
*Study 2a*			
CP->SC ->Expec. Neg. Treat.	.81	.23	0.45, 1.30*
CP->SC->Perc. Grp. Disc.	.76	.19	.42, 1.24*
*Study 2b*			
CP->SC->Expec. Neg. Treat.	-.33	.17	-.77, -.06*
CP->PP->Perc. Grp. Disc.	-.27	.18	-0.72, -.01*
*Study 2c*			
CP->PP->PI	.31	.14	.07, .64*
CP->PP->Expec. proc. just.	-.07	.08	-.28, .07
CP->PI->Expec. Proc. just.	-.16	.11	-.42, .03
CP->PP->PI->Expec. proc. just.	-.08	.05	-.20, -.01*
*Study 3a*			
CP->PP->PI	-.60	.25	-.17, -1.17*
CP->PI->Competence	-.23	-.13	-.02, -.56*
*Study 3b*			
Change in comp. -> threat to prototyp.->Grp. prej.	.53	.21	.20, 1.05*
*Study 4b*			
CP->SC->Commonality perceptions	-.24	.11	-.53, -.07*
*Study 5c*			
CP->PP->PI	-.52	.22	-.96, -.11*
CP->PI->Collective action	.05	.04	-.02, .16
CP->Efficacy->Collective action	1.01	.16	.71, 1.34*
CP->PI->Efficacy->Collective action	.07	.04	.01, .12*

*Note*. *CP* = Composition of place. *PP* = Place-prototypicality. PI = Place-identification. CI = confidence interval. “SC” refers to self-categorization at the level of social identity, “Expec. Neg. treat.” refers to expectations of negative group-based treatment, and “Perc. Grp. Disc. refers to perceived group discrimination. “Expect of proc just” refers to expectations of procedural justice, “PP” refers to place-prototypicality, and “PI” refers to place-identification for the respective spaces. “Will. to part. with L.E.” refers to willingness to partner with law enforcement. “Change in comp.” refers to change in composition of place, “threat to prototype.” refers to threat to prototypicality, and “Grp prej.” Refers to group prejudice. Scales ranged from 1 to 7.

*Indirect effect is reliable at *p* < .05 (excluding zero).

Study 2c compliments the findings of Studies 2a and 2b by illustrating that level of group representation of place also shapes expectations of fair treatment (procedural justice) in the respective place. In addition, the findings suggest that level of group representation of law enforcement agencies may be an initial antecedent associated with racial/ethnic minority group members’ willingness to partner with law enforcement. The results speak to the importance of diversity within law enforcement. Indeed, minority representation or diversity within law enforcement not only can have positive implications for officer behavior [e.g., reduce group-threat and office deadly use of force; 54], it can shape the way minority-group citizens engage with police [55]. The larger program of results suggest that diversity and proportionate representation is an integral aspect of bolstering the perceived legitimacy of criminal justice entities. Relative to a majority majority-group police force, vast and diverse representation of many groups should increase expectations of procedural justice, which is especially important for minority groups that often report less trust in authorities/law enforcement, compared to majority group members [56]. Study 2a-2c focused on individual approaching place, but it is expected that place-identification can also shape administrators of criminal justice [e.g., police officers and neighborhood identification; 57]. Studies 3a and 3b move beyond expectations to examine how place-identification and perceptions of prototypicality can impact the treatment of individual and group targets.

## Studies 3a & 3b: How level of group representation of place shapes individual- and group-level prejudice

Study 3a and 3b test the effect of relative level of group representation of place on individual-level bias (i.e., bias toward an individual target; Study 3a) and group-level prejudice (i.e., out-group prejudice; Study 3b), as explained by place-identification (Study 3a) and threat to place-prototypicality (Study 3b).

One potential implication of the present work, and more specifically the notion that there is variance in the degree to which people identify with places, is that it might be expected that people would show favoritism toward certain places (i.e., those in which they have high place-identification), over other places (i.e., low place-identification). From a social identity perspective, under conditions of salient social identity, group members often seek out strategies to achieve positive group differentiation [58], including showing favoritism toward those that share an inclusive identity [e.g., helping behavior; 59]. Thus, there is evidence to suggest that group members should show favoritism toward places in which they have higher place-identification, compared to places with relatively lower identification. Study 3a tests whether people demonstrate favoritism toward individual targets from places high in in-group representation, compared to those low in in-group representation, which was expected to be explained by place-identification.

### Study 3a

#### Method

Ninety-eight participants were originally recruited for the study, but five participants were dropped for failing the manipulation-check. The final sample was ninety-three self-identified White participants (48 men and 45 women; *M* age = 35.37).

The purpose of Study 3a was to put participants in the position to make evaluative and hiring judgements of an applicant. Therefore, the study was described as focused on “business decision-making” and designed to explore “how people make business evaluations regarding students.” All participants were given a two-page overview that described their position (“Head of Management and Evaluation”), the position that the company is hiring for (“An internship program. Looking to fill entry-level internship positions, which provide an opportunity for long-term employment”), and the selection process (“a holistic review, take into consideration both academic and non-academic achievements”). All of the descriptions were adapted from actual business internship programs online.

Next, participants were informed they would be evaluating one applicant named Conner Adams. Participants were instructed to assume they personally had knowledge about the general demographics of the University Conor Adams attended. That is, that “based on your knowledge of the University,” the demographics of the University were described as either predominantly “White” or “Black/African-American.” More specifically, a pie chart varied racial/ethnic demographic composition of the University, such that University was either predominantly White (75.10% Whites; 24.90% other racial/ethnic groups; i.e., high group-representation of Whites) or predominantly Black/African-American (75.10% Blacks/African-American; 24.90% other racial/ethnic groups; i.e., low group-representation of Whites).

On the next page, a manipulation-check assessed whether participants viewed the place as being low or high in representation of their group. Specifically, participants were asked ‘What is the racial/ethnic composition of the University?’, and answered on a 1 (*mostly non-White*) to 7 (*mostly White*) scale. On the following page, place-prototypicality (*α* = .89) was assessed, and on a subsequent page, place-identification (*α* = .90) with the University was measured. Next, all participants were given basic demographic information about the applicant under evaluation, including name (“Conner Adams”), gender (“male”), age (“22”), and race/ethnicity (“White”), as well as a resume for the applicant. Conner Adams’ resume indicated an average GPA (“3.4”) with both leadership (“Vice-President of Student government”) and business experience (“internship at Irvin & Smith accounting firm”). Thus, all participants were asked to evaluate the same White applicant (Conner Adams), but what differed was the demographic composition of his University.

Drawing on past work exploring evaluation of job applicants [60], the present research measured perceived competence, warmth, general work abilities, criteria for evaluation, hiring decision, and standards for promotion. More specifically, participants responded on 1 (*not at all*) to 7 (*extremely*) for two five-item scales assessing perceived competence (*capable*, *competent*, *organized*, *skillful*, and *intelligent*; *α* = .91) and warmth [*good-natured*, *sincere*, *warm*, *understanding*, and *kind*; *α* = .89; 61]. Next, participants were asked about the job applicant’s general work abilities using five items on a 1 (*less than the average applicant*) to 7 (*better than the average applicant*) scale (*α* = .86): “Motivation,” “Willingness to put in extra work (beyond what is expected),” “problem-solving,” “writing ability,” and “ability to accept instruction.”

On the next page, criteria for evaluation assessed information needed to make a final decision on the applicant. More specifically, criteria for evaluation assessed participants’ standards of evaluation and was designed to examine whether participants would utilize the same (or more) criteria for evaluation based on level of group representation. Participants were asked if they might need additional information to evaluate the applicant using three items (*α* = .71): “Additional letters of recommendation,” “Additional in-person interviews,” and “Additional in-person performance tests,” using 1 (*not at all necessary*) to 7 (*necessary*) scales. Decision to hire was assessed using two items (*r* = .88): “The applicant would make a very strong candidate for the position,” and “I would hire Conner Adams for the entry-level position,” on 1 (*strongly disagree*) to 7 (*strongly agree*) scales. Finally, participants were asked to assume they hired the applicant and told that one common practice among managers was to assign weaker applicants to “more hours/days to get more information to help make a promotion decision (i.e., from intern to employee).” Standards for promotion was assessed using three items (*α* = .83): “Hours per week” (1 = *less than 10 hours per week*, to 7 = *more than 25 hours per week*), “Days per week” (1 = *one day per week*, to 7 = *seven days per week*), and Days per month (1- *less than 10 days per month*, to, 7 = *more than 20 days per month*).

#### Results and discussion

Composition of place had a direct effect on perceptions of place-prototypicality, place-identification, and most of the applicant evaluation variables (see Table 6). For example, the applicant was perceived as having more competence under conditions in which the applicant came from a place with high group representation (*M* = 5.28, *SD* = 1.03), compared to under conditions of low group representation (*M* = 4.51, *SD* = 1.29), *t*(91) = 3.14, *p* < .01, *d* = 0.44, 95% CI = [.28, 1.25]. Moreover, participants used stricter criteria for evaluation under conditions when the target was from a space with low group representation (*M* = 4.80, *SD* = 1.25), compared to under conditions when the target was from a space with high group representation (*M* = 4.25, *SD* = 1.27), *t*(91) = -2.07, *p* < .05, *d* = 0.43, 95% CI = [.13, 1.21].

**Table 6:** Tests for the effect of group representation on place-prototypicality, place-identification, and intergroup outcome variables (Study 3a).

	Low group representation	High group representation			
	*M (SD)*	*M (SD)*	*t*	95%CI	*Cohen’s d*
Study 3a					
PP	3.68 (1.34)	5.09 (1.08)	5.54***	.90, 1.91	1.15
PI	3.60 (1.35)	4.69 (1.38)	3.86***	.53, 1.65	0.79
Competence	4.51 (1.29)	5.28 (1.03)	3.14**	.28, 1.25	0.44
Warmth	4.86 (1.12)	4.92 (1.01)	.259	-.38, .49	0.65
Work abilities	4.87 (1.23)	5.34 (.99)	2.08*	.022, .92	0.42
Evaluation criteria	4.80 (1.25)	4.25 (1.27)	-2.07*	-1.05, -.02	0.43
Hire	4.64 (1.29)	5.32 (1.33)	2.48*	.13, 1.21	0.51
Standards for promotion	3.34 (1.56)	4.03 (1.60)	2.12*	.04, 1.34	0.43

*Note*. *M* = Mean. *SD* = Standard Deviation. “PP” refers to place-prototypicality and “PI” refers to place-identification. Scales ranged from 1 to 7.

* *p* < .05.

** *p* < .01.

*** *p* < .001.

**Fig 11:**
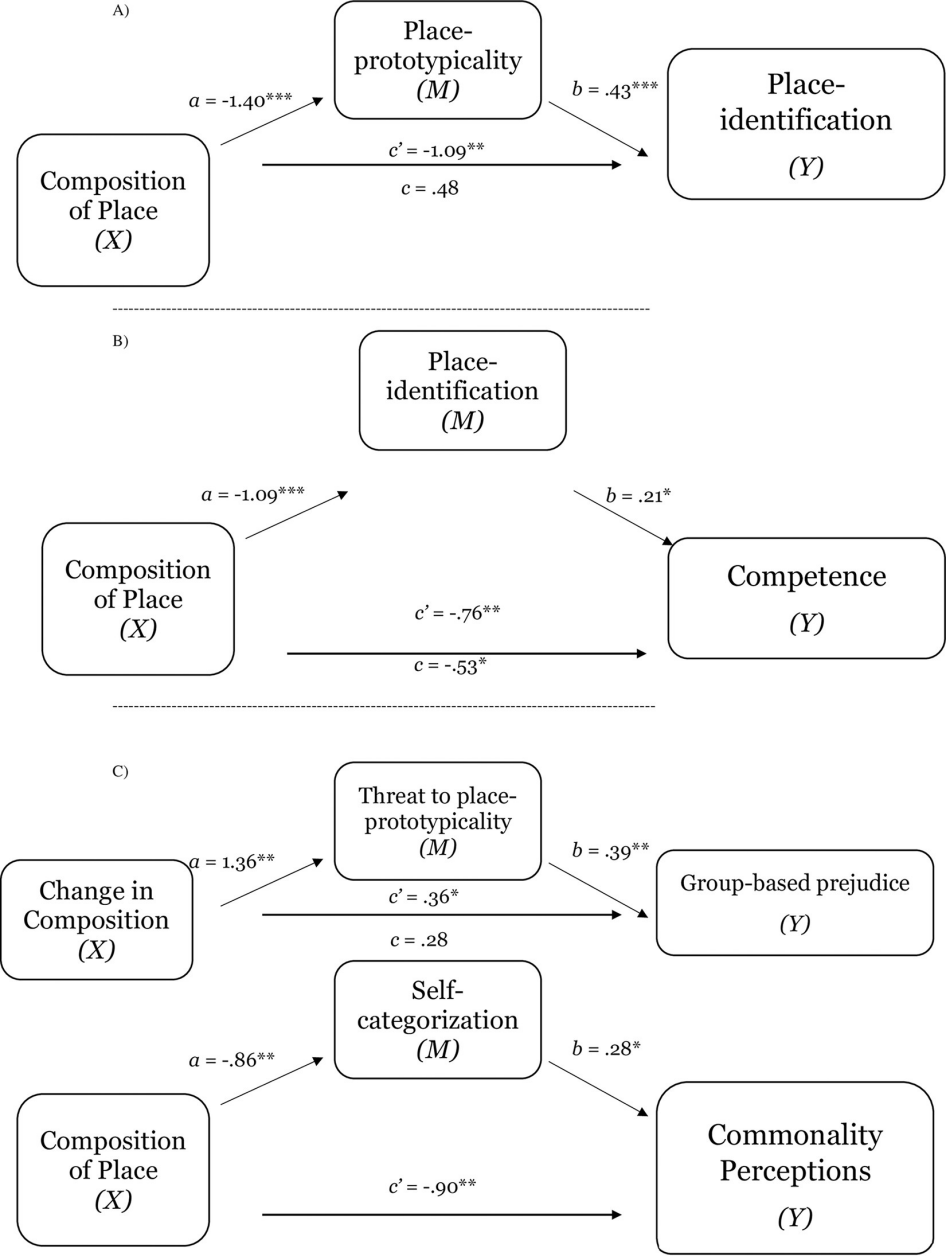
Study 3a mediation results. Test of the mediating effect of place-prototypicality on the relation between composition of place and place-identification (Panel A) and mediating effect of place-identification on the relation between composition of place and competence (Panel B). Test of the mediating effect of threat to place-prototypicality on the relation between change in composition of place and group-based prejudice in Study 3b (Panel C). Notes: *p < .05, **p < .01, ***p < .001.

Replicating the findings in the past studies, place-prototypicality explained the relation between composition of place and place-identification (see Table 5 and Fig 11, Panel A). Similarly, a test of the indirect effect of place-identification on the relation between composition of place and ratings of competence of the applicant was reliable (point estimate -.23, *SE* = .13; 95% CI = -.56 to -.02; see Fig 11, Panel B). The mediation tests (including sequential mediation) for the effect of composition of place on the other outcome variables were not significant or reliable.

The findings of Study 3a extend the present work by illustrating how composition of place can affect the treatment of targets of respective places. Participants used less stringent evaluation criteria, were more likely to view the applicant as competent, evaluated the target as having higher work abilities, and subsequently were more likely to hire the applicant under conditions when an applicant came from a high-group representation place, compared to when the applicant (with the same resume) came from a place with low group representation.

Although Study 3a provides evidence that place-identification explains the effects of composition of place on evaluative judgments of individual targets, the results did not find support that place-identification explained the effects of composition of place on perceptions of work abilities, evaluation criteria, or hiring decision. Recent work suggests that Whites view spaces predominantly composed of Black/African-American people as embodying stereotypes [i.e., space-focused stereotypes; 62], the failure of place-identification to explain the effects of composition of place on these respective outcomes may therefore be a function of space-focused stereotypes. The findings of Study 3a, though, complement and contribute to an emerging body of work on place-focused prejudice. Whereas past work suggests that places can embody the stereotypes of the dominant demographic group of the space [i.e., space-focused stereotypes; 62], the present work suggests that people may differentially evaluate objects and people derived from place as a function of identification with place.

More broadly, while it is expected that all group members should show a bias in favor of high group-representation places, the current findings (focused on majority-group members) are relevant for the maintenance of group-based inequality. Indeed, the findings suggest that majority group members may show an evaluative bias in favor of applicants from majority majority-group contexts. Thus, whether in the domain of evaluating applicants from schools or Universities (e.g., majority-minority; HBCU or HSI applicants) or from different neighborhoods (i.e., majority majority-group versus majority minority-group), the findings of Study 3a suggest that majority-group members may implicitly favor targets from majority-group places. Whereas Study 3a focused on how composition of place shapes bias toward individual-level targets, Study 3b examines how feeling threatened about the predominant values of place can explain group-level prejudice.

Study 3b examines responses to shifts in group-based perceptions of place, with a focus on threat to place-prototypicality: the feeling elicited from events that challenge the perceived predominant values or characteristics of a respective place. More specifically, the study examined how threat to place-prototypicality may explain majority-group prejudice against minority groups’ in contexts where there are shifts in composition indicate greater minority-group representation. Separate programs of work simultaneously demonstrate that increases in minority group representation are associated with increased prejudice among minority groups [63–65], but also that threat to perceived values increases bias against minority groups [66,67]. Indeed, concerns about shifts in cultural values has been shown to explain majority group members’ prejudice toward Muslims [e.g., in the Netherlands; 67] and immigrant groups [68]. To the extent that there is a direct relation between representation of one’s group and perceptions that a place is representative of one’s group unique values (as indicated in the results of the previous studies), increases in minority group representation/decreases in majority group representation should elicit threat to place-prototypicality. Study 3b investigates whether shifts in composition of place are associated with increased majority-group prejudice toward a minority group, which was expected to be explained by perceived threat to the prototypicality of the respective place.

### Study 3b

#### Method

One hundred and two self-identified White Christian/Catholic participants (62 women and 40 men; *M* age = 39.03), a racial/ethnic and religious majority group in the United States, participated in the study. The purpose of the study was described as exploring “social attitudes” and an investigation of “perceptions of the people of the United States and policy attitudes.” Participants were randomly assigned to a condition that emphasized that the religious make-up of the United States would either stay the same (*no-change in composition condition*) or change (*change in composition condition*) by 2027 (ten years after time of the study). More specifically, all participants were given a pie chart reporting the 2017 (current at time of study) religious demographics of the United States: 72% Catholic/Christian, 22% Atheist/unaffiliated, 3% Jewish, 2% other, and 1% Muslim. Directly under the 2017 information, a second pie chart provided estimates regarding the expected religious make-up of the country in 2027. In the *No-change in composition* condition, the second pie chart indicated no change in the religious composition of the United States in 2027: 72% Catholic/Christian, 22% Atheist/unaffiliated, 3% Jewish, 2% other, and 1% Muslim. In addition, a short message under the chart read (in bold) “the religious make-up of the country is expected to stay the same.” In the *Change in composition* condition, the second pie chart indicated there would be a simultaneous decrease of Christians/Catholics and increase of Muslims in the United States by 2027: 51% Catholic/Christian, 24% Atheist/unaffiliated, 20% Muslim, 3% Jewish, and 2% other. In addition, a short message under the chart read (in bold) “there will be a decrease in Catholics/Christians and an increase in Muslims.” Thus, participants in both conditions were given information about the religious make-up of the United States in 2017 and 2027, but only in the *change in composition* condition did majority group participants receive information of a decrease in majority-group representation (increase in minority-group representation). The format for this manipulation was adapted from past work exploring demographic change and group-based prejudice [63]. On the next page, two questions served as a manipulation-check assessing participants’ perception of religious composition change in 2027: (a) “What is the expected religious composition of the United States in 2027?”, answered on a 1 (*Less Christians/Catholics*), 4 (*About the same*), and 7 (*More Catholics/Christians*) scale and (b) What is the expected religious composition of the United States in 2027?”, answered on a 1 (*Less Muslims*), 4 (*About the same*), and 7 (*More Muslims*) scale.

On the following page, participants were asked to “think about the expected religious make-up of the United States in 2027.” Threat to place-prototypicality was assessed by asking participants if they perceived that changes in the religious make-up of the country would make the country less “representative of the unique values and beliefs” that define their religious group or orientation. More specifically, participants were asked: “How worried would you be that the United States would not…”: “…represent what is characteristic of Christians/Catholics”, “…be representative of the unique values of Christians/Catholics,” and “…exemplify the beliefs that define Christians/Catholics. (*α* = .95).

Finally, on the last page among a variety of filler items, participants indicated support for a number of policies targeting the minority group: the Muslim community. That is, participants were asked whether the United States should “adopt more aggressive policies regarding Muslims and limit the number of Muslims in the country.” More specifically, participants were asked “…given the projections you read about earlier, how supportive would you be of the United States implementing the following policies now?” Four items assessed support for Anti-Muslim policies [69]: “Create a ‘Muslim ban’ for immigration for all people from Muslim-majority countries,” “Limit the number of people the United States accepts from Muslim countries,” “Create a Muslim registration list, requiring that all Muslims register with the U.S. government,” “Create a ‘Muslim enhanced interrogation’ technique policy, such that law enforcement would be allowed to use enhanced interrogation techniques on Muslims.” Participants responded on a 1 (*strongly oppose*) to 7 (*strongly support*) scale (*α* = .91).

#### Results and discussion

Consistent with hypotheses, change in composition was associated with increases in threat to place-prototypicality and support for aggressive or discriminatory policies targeting the minority group. More specifically, there was an effect of experimental condition on threat to place-prototypicality, *t*(100) = -3.96, *p* < .001, *d* = -0.78, 95% CI = [-.2.05, -.68]. Participants in the *change in composition* condition (*M* = 4.60, *SD* = 1.76) reported higher threat to place-prototypicality, compared to those in the *no-change in composition* condition (*M* = 3.23, *SD* = 1.72). Similarly, there was an effect of experimental condition on support for Anti-Muslim policies, *t*(100) = -2.30, *p* < .001, *d* = -0.46, 95% CI = [-.1.53, -.11]. Participants in the *change in composition* condition (*M* = 4.41, *SD* = 1.87) reported higher support for the implementation of Anti-Muslim policies, compared to those in the *no-change in composition* condition (*M* = 3.58, *SD* = 1.72). A test of the indirect effect of threat to place-prototypicality on the relation between experimental condition and support for Anti-Muslim policies was reliable (threat to place-prototypicality; estimate for the indirect effect was .53, *SE* = .21; 95% CI = .20 to 1.05; see Fig 11, Panel C).

Complimenting classic [70] and contemporary [63] work, Study 3b provides additional evidence that level of group representation of place has implications for the study of prejudice. Importantly, the findings locate concerns about the group-based nature of place, in particular the characteristics that make the group unique from other groups, as noteworthy for responses to shifts in composition of place. Indeed, to the extent that group members view an existing place as prototypical of their group—they are likely to exhibit group-based prejudice when they perceive this prototypicality as being threatened. Thus, majority group concerns about the extent to which a place will be representative of the unique values of the respective majority group may help to explain majority-group bias, prejudice, and aggression toward minority groups. In line with Mill [71] and Tocqueville [72] reasoning on the topic of the *tyranny of the majority*, there is often a danger in direct democracy if or when majority groups place their own interests above or at the expense of a minority or minority groups. Given that the 2027 projections in the change in composition condition in the current study indicated that Catholics/Christian would *still* be a majority group (i.e., 51% of the nation), the findings are especially poignant. The findings of Study 3b demonstrate that majority group behavior toward minority groups may be dependent on composition of place of the respective context and illustrate the need to acknowledge or prioritize the social context of intergroup relations in analyzing and understanding the expression of prejudice.

More generally, Studies 3a and 3b complement a growing body of work that provides a context-driven account of prejudice. Emerging research suggests that rather than conceiving of prejudice as a universal, it is much more accurate to view prejudice and bias as a dynamic outcome—often dependent on context. For example, prejudice in the form of automatic evaluative responses is often dependent on social context [73,74]. Similarly, several studies illustrate that data derived from WEIRD [Western, educated, industrialized, rich, democratic; 75] samples do not always generalize to other cultures, demonstrating the role of cultural context in the expression of prejudice [76]. Thus, the findings of Study 3a and 3b suggest that composition of place is one such contextual factor that may shape motivation and expression of prejudice.

The program of research, thus far, suggests that composition of place, perceptions of place-prototypicality, and sense of belonging have direct implications for expectations of treatment within place, attributions for group-based events, and bias or prejudice toward both individual and group targets. Providing further evidence that composition of place shapes a variety of collective behavioral tendencies, the remaining studies examine how composition of place shapes outcomes relevant to social justice and the amelioration of inequality.

## Studies 4a-4c: Level of group representation within place and the efficacy of prejudice-reduction interventions, solidarity, and collective action

The final set of studies were designed to demonstrate the role of composition of place in shaping the efficacy prejudice-reduction intervention (Study 4a), political solidarity among minority groups (Study 4b), and collective action (Study 4c). Study 4a explores whether the efficacy of interventions to improve intergroup attitudes may be dependent on composition of place. A large and robust literature demonstrates that emphasizing commonality, or altering the perception of group boundaries, is associated with more positive intergroup attitudes, greater empathy toward out-group targets, and greater propensity to engage in pro-social behaviors toward out-groups [77]. However, past work demonstrates that context can moderate the efficacy of commonality-based bias-reduction interventions, such that shared context [78], out-group characteristics expressed in context [79], or intergroup contact with other groups [80] shape the efficacy of bias-reduction interventions. It is more effective to emphasize commonality, for example, under conditions where groups share a context, compared to under conditions of separate context [78]. In line with these findings, it was hypothesized that composition of place would moderate the effect of a commonality-focused bias reduction intervention on out-group attitudes, such that as level of group representation increases, the efficacy of the intervention would improve, which would be explained by identification with place.

### Study 4a

#### Method

Two hundred and fifty-six participants self-identified Latinx (Latina/Hispanic; 181 women and 75 men; *M* age = 23.24) participated to fulfill one option of an introductory psychology course. Participants individually completed questionnaires in a group setting. The design for the study was a 2 (Commonality identity emphasis: *Commonality vs*. *control*) x 3 (Composition of space: *Majority-White*, *Integrated-Mixed*, *and Majority-Latino space*) factorial design.

Participants were informed that the researchers were interested in forming “discussion groups between people of different racial/ethnic groups” to help “groups understand one another and build bridges across racial/ethnic divides.” For the initial commonality identity-emphasis condition, participants were randomly assigned to one of two conditions: commonality or control [method for manipulation adapted from 81]. In both conditions, participants were asked to “consider the following information when considering the discussion group” and read a general paragraph describing diversity in America, including the following passages: “The population of the United States includes many different racial/ethnic groups…As our country becomes more diverse and begins to include many groups, it becomes critical to understand how group think about and relate to one another.” This initial generic paragraph served as the baseline comparison condition (control condition). In the commonality condition, participants read an additional paragraph designed to blur intergroup boundaries between Latinx (Hispanics) and White racial/ethnic groups. In the commonality condition, the paragraph read, in part, “Experts from different fields have recognized that Latinas (Hispanics) and non-Hispanic Whites (Caucasians) share a common identity in the sense that they both share basic values rooted in a common national identity (American)…it is agreed that each group could benefit from thinking more in terms of common national identity…Thus, social scientists have confirmed the existence of a common group identity..”

On the following page, all participants were then given a biased response-format, which further emphasized the goal of each condition [80,82]. More specifically, in the commonality condition, participants were asked to complete three tasks: (a) write down five reasons why “Latino/as and Whites share a common identity”, (b) choose a statement from four (similarly-worded) options that best summarized the news report from the previous page (e.g., “Latino/as and Whites should emphasize a shared identity, not racial/ethnic divisions), and finally (c) choose a pictorial representation from two pictures depicting the “relations between Latino/as and Whites.” Each pictorial representation had two circles along a continuum of distance, Latino/as represented by a circle on one side and the other circle representing Whites on the other end of the continuum [83]. In the commonality condition, the biased choice was between two representations that depicted Latinos and Whites as semi-overlapping: each of the circles overlapped with each other, suggesting commonality. In the control condition, participants completed the following three tasks: (a) write down five reasons why there are a number of groups in America (b) choose a statement from four (similarly-worded) options that best summarized the news report from the previous page, (e.g., “the population includes many groups”) and finally (c) choose a pictorial representation from two pictures depicting the “relations between Latino/as and Whites.” In the control condition, the pictorial representation depicted two circles far apart with only slight overlap between circles. In all conditions, participants responded to a manipulation check item on the next page: “I view Latino/as/Hispanics and Whites as a part of ONE group (American)” on a 1 (*strongly disagree*) to 7 (*strongly agree*) scale.

Next, composition of place was manipulated by varying the expected composition of place for the discussion group. Specifically, participants again informed that the researchers of the study were conducting “discussion groups between different racial/ethnic groups…focused on positive relations and building bridges,” that the discussion group would occur “off-campus, but within walking distance,” and that “based on sign-ups and space-constraints the composition of these discussion groups would likely vary.” For the composition of place factor, participants were randomly assigned to one of three space conditions: *Majority-White*, *Integrated-Mixed*, and *Majority-Latinx*. In the *Majority-White* condition, participants read a short paragraph informing them of the composition of place for the discussion group: “…based on the number of people that have already signed up, it is expected the discussion group will occur in a space composed of predominantly non-Hispanic White (Caucasian) people” and that “…the majority of people at the discussion group will be White,” such that there will be “…significantly more White people at the discussion group, compared to Hispanic/Latino people.” In the *mixed-integrated* condition, participants were informed that it was expected that there would “a mix of races/ethnicities, likely about half Latino/a/Hispanic people and half White/Caucasian people” In the *Majority-Latinx* condition, participants read a short paragraph informed that: “…based on the number of people that have already signed up, it is expected the discussion group will occur in a place composed of predominantly Latino/a/Hispanic people” and that “…the majority of people at the discussion group will be Latino/a/Hispanic,” such that there will be “…significantly more Hispanic/Latino/a people at the discussion group, compared to White/Caucasian people.” Thus, Study 4a provides a test of the effect of composition of place utilizing an alternative means of manipulating level of group representation (text versus pie-charts in previous studies).

On the next page, place-identification (*α* = .92) was assessed for the discussion group. In addition, participants were also asked to report the level of anxiety for the upcoming discussion using three items [adapted from 84; *uneasy*; *anxious*; *uncomfortable*; *α* = .88] on a 1 (*does not apply*) to 7 (*applies very much*) scale. Next, attitudes toward Whites was assessed via a standard thermometer scale [e.g., 85], in which participants were asked to ‘describe their feelings toward Whites at the moment’, ranging from 0 *(Cold*) to 100 (*Warm*). Finally, two items assessed participants’ decision to attend the discussion group, with participants responding on a 1 (*strongly disagree*) to 7 (*strongly agree*) scale (*r* = .90): “If I had the choice, I would go to the discussion group” and “I would like to go to the discussion group.”

#### Results and discussion

A 2 (Identity emphasis: Commonality vs. Control) x 3 (Composition of place: low, equal, or high group-representation) two-way factorial ANOVA was conducted for each of the outcome variables. Table 7 (results of ANOVA) and Table 8 (means, standard deviations) report the results for all outcome variables.

**Table 7:** Identity-emphasis x group representation analysis of variance for outcome variables (Study 4a).

	*df*	*F*	*η* ^2^_*p*_
*Place-Identification*			
Ident. emphasis (A)	1	4.08*	.016
Comp. of place (B)	2	48.99***	.276
A x B (Interaction)	2	.101	.001
Error	250		
*Anxiety*			
Ident. emphasis (A)	1	7.59**	.030
Comp. of place (B)	2	22.84***	.157
A x B (Interaction)	2	3.99*	.032
Error	245		
*Out-group attitudes*			
Ident. emphasis (A)	1	8.59**	.037
Comp. of place (B)	2	4.80**	.041
A x B (Interaction)	2	2.84^†^	.025
Error	223		
*Decision to attend discussion with out-group*			
Ident. emphasis (A)	1	.73	.003
Comp. of place (B)	2	2.27	.018
A x B (Interaction)	2	7.46**	.057
Error	248		

*Note*. *“Ident*. *Emphasis” refers to identity emphasis condition (i*.*e*., *Commonality vs*. *control)*.

^†^ < .07

* *p* < .05.

** *p* < .01.

*** *p* < .001.

**Table 8:** Tests for the effects of identity emphasis and group representation on outcome variables (Study 4a).

	Composition Condition		
	Low group representation (minority status)	Mixed (Equal representation)	High group representation (majority status)
*Place Identification*			
Commonality condition	3.54 (1.40)_a_	4.65 (1.21)_b_	5.58 (1.21)_c_
Control condition	3.18 (1.52)_a_	4.41 (1.63)_b_	5.15 (1.21)_c_
*Anxiety*			
Commonality condition	3.35 (1.60)_a_	3.38 (.99) _c_	2.20 (1.17)_d_
Control condition	4.27 (1.13)_b_	3.14 (1.42)_c_	2.76 (1.38)_d_
*Out-group Attitudes*			
Commonality condition	59.30 (30.80) _a_	60.40 (30.03) _a_	77.10 (22.61) _b_
Control condition	51.30 (16.54) _a_	60.41 (25.31) _a_	56.60 (30.21) _a_
*Decision to attend discussion with out-group*			
Commonality condition	3.34 (1.69) _a_	4.20 (1.40) _b_	4.72 (1.98) _c_
Control condition	4.20 (1.73) _a_	3.78 (1.96) _b_	3.61 (1.33) _b_

*Note*. †< .07, * *p* < .05, ** *p* < .01, *** *p* < .05. Scales ranged from 1 to 7. Means (standard deviations) appear in table. Higher scores indicate greater place-identification, anxiety, positive out-group attitudes, and willingness to attend a discussion with an out-group. Means with differing subscripts are significantly different at the *p* < .05 level based on planned post-hoc paired comparisons. For place-identification, subscripts indicate differences pairwise comparisons across composition of place conditions. For anxiety, out-group attitudes, and decision to attend discussion group, subscripts indicate differences in simple main effects of identity emphasis condition within the respective composition of place conditions.

For place-identification, post-hoc tests to compute pairwise comparisons for the two main effects of identity emphasis and composition of place, in which Bonferroni adjustment was applied, revealed that all pairwise comparisons were significant, *p* < .001. For anxiety, the two main effects were qualified by a significant interaction. Post-hoc tests were performed to analyzed the simple main effects of identity emphasis for each condition of composition of place with statistical significance receiving Bonferroni adjustment, which revealed that only the low-representation condition (majority-White) was statistically significant, *F*(1, 245), 11.20, *p* < .001, *η*^2^_*p =*_ .04. Thus, under conditions of low representation, the commonality intervention reduced anxiety, relative to the control. For intergroup attitudes, the two main effects were also qualified by a marginal two-way interaction. Post-hoc tests to analyze the simple main effects revealed significant difference only in the high group-representation condition, *F* (1, 223) = 12.30, *p* < .001, *η*^2^_*p =*_ .05. Under conditions of high-group representation, participants reported more positive out-group attitudes in the commonality condition, compared to the control. Neither of the main effects were significant, but the interaction was reliable. Post-hoc tests to analyze the simple main effects for identity emphasis for each composition of place condition with statistical significance receiving Bonferroni adjustment were conducted, which revealed significance for both the low-representation (majority-White) condition, *F*(1, 248) = 5.71, *p* < .05, *η*^2^_*p =*_ .02, and the high group-representation condition (majority-Latinx), *F* (1, 248) = 8.85, *p* < .01 *η*^2^_*p =*_ .03. Under conditions of high-group representation, participants, for example, were more likely to decide to attend the discussion group in the commonality condition, compared to the control. Conversely, under low representation conditions (majority-White), participants were less likely to decide to attend the discussion group in the commonality condition, compared to the control.

The findings of Study 4a provide initial evidence of the role of composition of place in shaping the efficacy of prejudice-reduction interventions. Indeed, the efficacy of emphasizing commonality (relative to control) was dependent on level of group representation of place for anxiety, out-group attitudes, and decision to join a discussion group (though with distinct patterns). The findings suggest that relative level of group representation of place may shape prejudice-reduction outcomes, but also may have implications for explaining the divergent effects of prejudice-reduction interventions on minority and majority-group members, respectively.

A large body of work demonstrates that prejudice-reduction interventions, such as contact and common identity, are often less effective at improving attitudes for minority group members, compared to majority group members [86–88]. Much of the work explaining divergent outcomes of prejudice-reduction interventions between minority and majority group members has focused on personal or intergroup factors, such as the content of discussion [89], differences in the potential for positive contact outcomes [88], or distinct affective experiences [90]. The results of Study 4a, however, suggest that the efficacy of prejudice-reduction interventions may also be dependent on composition of place or intergroup context. Indeed, to the extent that most prejudice-reduction interventions occur in majority majority-group contexts, the divergent outcomes of majority and minority- group members may be partially a function of distinct responses to respective level of group-representation within place and the role of place in moderating the efficacy of prejudice-reduction intervention on factors relevant to out-group attitudes (e.g., anxiety).

One intriguing pattern of findings is how composition of place, place-identification, and anxiety manifest under conditions of commonality. For example, across all conditions there was a negative association between place-identification and anxiety (*r* = -.44, *p* < .001), but under conditions of commonality with a high-level of in-group representation there was less strong association between the two constructs (*r* = -.18, *p* = .19). Generally, it would be expected that place-identification is associated with less anxiety (consistent with the correlation across conditions), but if some of the anxiety associated with prejudice-reduction interventions for minority group members is rooted in the experience of being in a majority-group context, there may be less anxiety and (as such) less variance to be explained under conditions of relative high-group representation. The present findings, though, suggest that one fruitful avenue for future work concerns the interrelation among composition of place, place-identification, anxiety and the efficacy of prejudice-reduction interventions. Whereas Study 4a was designed to explore intergroup attitudes, Study 4b examines how level of group representation within place affects another construct relevant to social justice and the amelioration of group inequality.

Study 4b investigates the implications of composition of place for solidarity among minority groups. Studies 2a and 2b provide evidence that level of group representation has a direct effect on the self-categorization at the level of social identity, such that group members are more likely to categorize in terms of a social identity under conditions of low group-representation. Past work finds that the extent to which minority group members think in terms of racial/ethnic group membership (e.g., perceived discrimination) is associated with support and solidarity on behalf of another minority group [91]. It was expected that under conditions of low group representation, minority group members would report higher solidarity with another minority group, compared to under less group representation, which would be explained by increased tendency to self-categorize in terms of their racial/ethnic minority group membership.

### Study 4b

#### Method

One hundred self-identified Black/African-American participants (51 men and 49 women; *M* age = 33.24) participated in Study 4b. Participants were instructed to ‘imagine they had been accepted to a University’ and told the purpose of the study was to understand how people ‘respond to everyday events on campus.’ All participants were given one page of information describing a number of characteristics of the University, including student-faculty ratio, commitment to small classrooms, desire to build a close-knit community, and provide students with hands-on experience in the lab and in the field. A second page provided information about the demographic composition of the entire University with respect to race/ethnicity in a pie chart and varied representation of the group, such that there was either a low (24.90% other racial/ethnic groups, 75.10% Whites) or a high representation of Blacks/African-Americans (75.10% other racial/ethnic groups; 24.90% White). Thus, the manipulation was designed to imply relatively greater group representation, but not necessarily majority-group status for Blacks/African-Americans in the ‘high representation’ condition (i.e., conditions contrasted *majority minority-group* vs. *majority majority-group*) On the next page, two manipulation-check items assessed participants perceptions of the representation of place. Specifically, participants were asked ‘What is the racial/ethnic composition of the University?’, and answered on a 1 (*mostly non-White*) to 7 (*mostly White*) scale. In addition, participants were asked “Consider the University, would you expect there to be many Black/African-American students on campus?,” and answered on a 1 (*not many Black/African-American students*) to 7 (*many Black/African-American students*) scale.

On the following page, participants were instructed to consider living on campus, walking around the University, and interacting with others students. Self-categorization was assessed using the same items as Study 2a (*α* = .81). To provide an additional, more nuanced, evidence of shift in self-categorization, from subordinate level (personal identity) to intermediate level (social identity), Study 4b also included an additional item assessing self-categorization [92]: “How would you think about yourself on campus?” (1 = *as a unique person*, to, 7 = *as a part of my racial/ethnic group*). On the next page, perceptions of commonality with Latinas were assessed using three items [80]: “Blacks and Latino/as (Hispanics) would have similar experiences on campus,” “Blacks and Latino/as (Hispanics) would likely share common experiences on campus,” and “Blacks and Latino/as (Hispanics) would be likely to have common values on this particular campus.” Participants responded on a 1 (*strongly disagree*) to 7 (*strongly agree*) scale (*α* = .88). Next, motivation for contact with the other minority group was assessed using three items: “I would be motivated to make friends with Latinos/Hispanics on campus,” “I would seek out Latinos/Hispanics on campus to become friends,” and “I would be especially likely to make friends with Latinos/Hispanics on campus.” Participants responded on a 1 (*not at all likely*) to 7 (*very likely*) scale (*α* = .80). Finally, on the last set of pages assessed political solidarity.

Finally, preference for political solidarity was assessed. Participants were asked to imagine that there had been several incidents of ant-Hispanic/Latino discrimination on campus (e.g., graffiti and an assault of one Latino student), many of which likely came from groups outside of the campus. Participants were asked how likely they would be to engage in collective action on behalf of Latinos/Hispanics using three items [93]: “If given the opportunity, I would participate in a protest on behalf of Latinos/Hispanics on campus,” “I would participate in events to raise awareness about injustices faced by Latinos/Hispanics on campus, “If given the opportunity, I would sign a petition to improve security to help protect Latino/Hispanic students.” Participants responded on a 1 (*extremely unlikely*) to 7 (*extremely likely*) scale (*α* = .85).

#### Results and discussion

There was a direct effect of composition of place on self-categorization, using the original measure of self-categorization, *t*(98) = 3.04, *p* < .01, *d* = 0.63, 95% CI = [.29, 1.42], as well as the more nuanced measure of self-categorization, *t*(98) = 2.15, *p* < .05, *d* = 0.44, 95% CI = [.06, 1.46]. Consistent with previous results, participants were more likely to self-categorize in terms of their racial/ethnic group membership (i.e., at the level of social identity) under conditions of low group-representation (*M* = 4.87, *SD* = 1.08; *M* = 4.64, *SD* = 1.57), compared to under conditions of relatively greater group-representation (*M* = 4.01, *SD* = 1.60; *M* = 3.88, *SD* = 1.87).

**Fig 12:**
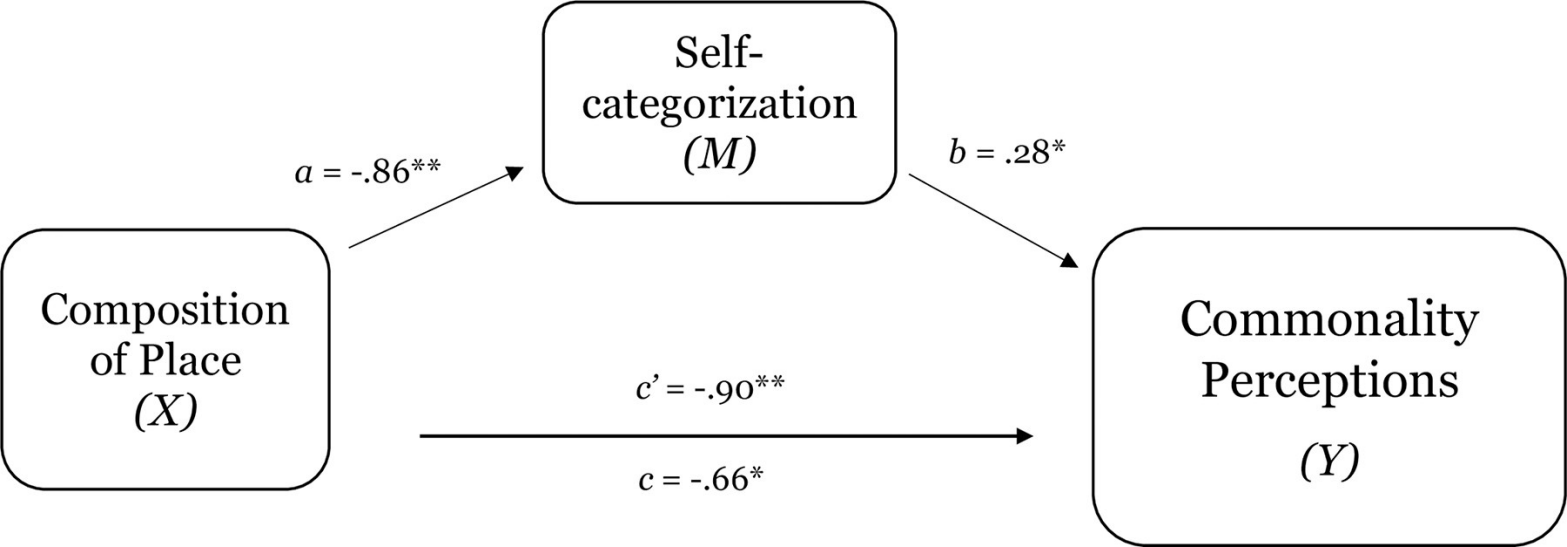
Test of the mediating effect of self-categorization on the relation between composition of place and perceptions of commonality in Study 4b. Notes: *p < .05, **p < .01, ***p < .001.

Level of group representation within place also had an effect on perceptions of commonality and motivation for contact with another minority group. Specifically, under conditions of low group-representation (*M*_**comm**_ = 5.03, *SD* = 1.05; *M*_**motiv**_ = 5.01, *SD* = 1.12), participants were more likely to perceive commonalities with another minority group, *t*(98) = 3.52, *p* < .001, *d* = 0.72, 95% CI = [.39, 1.41], and report a higher motivation for contact with another minority group, *t*(98) = 3.04, *p* < .01, *d* = 0.61, 95% CI = [.26, 1.25], compared to under conditions of relatively greater group-representation (*M*_**comm**_ = 4.13, *SD* = 1.42; *M*_**motiv**_ = 4.25, *SD* = 1.31). However, there were no differences between the representation conditions for collective action on behalf of another minority group, *t*(98) = .96, *p* = .33. A test to explore whether self-categorization (original measure) explained the effects of composition of place on perceptions of commonality was reliable (estimate -.24, SE = .11; 95% CI = -.53 to -.07; see Fig 12). These effects were replicated using the second measure of self-categorization, which assessed shift from subordinate level (personal identity) to intermediate level (social identity; estimate -.17, *SE* = .09; 95% CI = -.41 to -.02). The test of the indirect effect of self-categorization on motivation for contact, however, was not significant (CI = -.20 to .12).

Study 4b suggests that composition of place can shape solidarity among minority groups. Much of the work on relations among minority groups suggests that perception of a shared experience of disadvantage (e.g., similar struggles with being the target of prejudice) can improve relations among minority or stigmatized groups [94]. The findings of Study 4b suggest, however, the place or context of intergroup relations may moderate the efficacy of a ‘shared disadvantage’ approach. The manipulation of Study 4b suggested relative greater representation, but not necessarily majority-group status, providing additional evidence that level of group representation and not majority-group status, *per se*, is associated with collective outcomes. Indeed, under conditions of relatively greater group-representation, minority group members were less likely to categorize in terms of their specific minority social identity and (in turn) less likely to view commonalities with another minority group. The findings have implications for a ‘shared disadvantage’ approach to minority solidarity [94]. It is possible that the meaning of shared interest or shared disadvantage changes under differing conditions of composition of place (e.g., predominantly White suburb vs. racially diverse city), such numbers increase for a specific minority-group relative to the total composition, group members may advocate for more numerous, diverse, or nuanced interests for their own group—at the expense of shared interest with other disadvantage groups. Future work is, of course, needed to more fully explore the way demographic composition may influence relations among minority groups, but the present work provides clear evidence of the effects of composition of place, as well as self-categorization, on shaping perceptions of commonality.

More broadly, the findings of Study 4b point to the importance of acknowledging context in the study of minority-minority relations. The default (often unacknowledged) assumption of many studies is a majority-group context (e.g., predominantly White), but (as demonstrated by the findings in the current work), changing the composition of context can lead to a change in patterns of intergroup dynamics. Thus, it should not be assumed that findings regarding inter-minority relations that have been studied within predominantly majority-group contexts will necessarily generalize to other group-composition contexts (e.g., predominantly minority-group). Moreover, it seems reasonable to note that studying inter-minority relations within predominantly majority-group contexts may also produce narratives of solidarity (e.g., among Black and Latinx) that may not exist in alternative contexts (e.g., predominantly Latinx or Black contexts). Indeed, the current findings would suggest that as demographics change within a context [e.g., in the United States; 63], potential for conflict among minority groups may also increase. Thus, consistent with calls to move beyond the ‘two-group paradigm’ within intergroup relations empirical studies [95], the findings of Study 4b illustrate that data collection that assumes a predominant-group context may lead to a narrow set of conclusions that do not necessarily generalize to all contexts. Having demonstrated that the implications of composition of place for two outcomes relevant to amelioration of inequality (efficacy of prejudice-reduction and solidarity), Study 4c examined the effect of level of group representation of place on willingness to work on behalf of one’s own group (collective action).

Study 4c tests whether level of group representation within place affects minority group collective action tendencies. Past work demonstrates that there is a positive association between social support and group-efficacy, such that to the extent that group members perceive they have social support (e.g., greater numbers), they are more likely to report group-efficacy [96]. Group efficacy, in turn, is often associated with collective action tendencies [97]. Taken together, these findings suggest that level of group representation should have a direct effect on collective action tendencies, which should be explained by group-efficacy.

### Study 4c

#### Method

One hundred and fifty participants self-identified Black/African-American participants (97 women and 53 men; *M* age = 34.75) participated in Study 4c. Participants were instructed to ‘imagine they had been accepted to a University’ and told the purpose of the study was to understand how people ‘respond to everyday events on campus.’ All participants were given a one page of background information about the University. A second page provided information about the demographic composition of the entire University in a pie chart and varied representation of the group, such that there was either low representation (18.90% other racial/ethnic groups, 81.10% White) or relatively greater representation of Blacks/African-Americans (38.90% other racial/ethnic groups; 61.10% White). Thus, in both conditions the manipulation suggested Blacks were a minority-group, but one condition suggested greater relative group representation of the minority group. On the next page, two manipulation-check items assessed perceptions of group-representation. Specifically, the first manipulation-check item asked participants ‘What is the racial/ethnic composition of the University?’, and respondents answered on a 1 (*mostly White*) to 7 (*mostly non-White*) scale. In addition, a second manipulation-check item asked participants, “Consider the University, would you expect there to be many Black/African-American students on campus?,” and answered on a 1 (*not many Black/African-American students*) to 7 (*many Black/African-American students*) scale.

On the following page, participants were instructed to consider living on campus, walking around the University, and interacting with others students. Perceptions of place-prototypicality (*α* = .92) and place-identification (*α* = .96) were assessed using identical items to past studies. On the next page, participants were asked to imagine a group had invited a speaker that “some students on campus viewed as controversial or threatening” for his views on Blacks/African-Americans. Participants were provided with a list of quotes from recent speeches and books, including the following: “It’s difficult for Black people to submit to White leadership,” “If you look at history, Black people are so violent, they haven’t evolved yet,” “If you think about the future, I think White people need to be careful with intermarriage with Blacks,” “I call on White volunteers to rise up and be prepared for violence in the face of threat of Black people,” and “Anything negative that comes to Black people, they deserve it.” Participants were then informed that because of the invitation of this speaker, Black/African-American students on campus had organized to advocate that the University review policies and procedures regarding speaker invitations.

On the following page, perceived group-efficacy regarding Black/African-American students’ ability to successfully change University policy was assessed. Participants were asked: “…to what extent could you, working with other Black/African-American students on campus, advocate to successfully change the speaker-invitation policies of the University?” Group-efficacy was measured using four items: “Working with other Black/African-American students, I think we could change the speaker-invitation policies of the University,” “I think working together we could change this situation,” “Black/African-American students, working together, could successfully stand up for their rights on campus,” and “Black/African-American students could influence the decision of the University officials regarding speaker-invitation policies.” Participants answers on a 1 (*not at all likely*) to 7 (*very likely*) scale (*α* = .96) and the items were adapted from past work [98]. Finally, willingness to engage in collective action was assessed using four items: “I would sign a petition advocating to review speaker-invitation policies,” “If I had the opportunity, I would take time out of my day to participate in a demonstration against discrimination against Blacks/African-Americans on campus,” “In support of Blacks/African-Americans, I would engage in direct action to pressure the University to review speaker-invitation policies,” and “Participate in a panel discussion to promote change in speaker-policies on campus.” Participants responded on a 1 (*not at all likely*) to 7 (*very likely*) scale (*α* = .90).

#### Results and discussion

**Fig 13:**
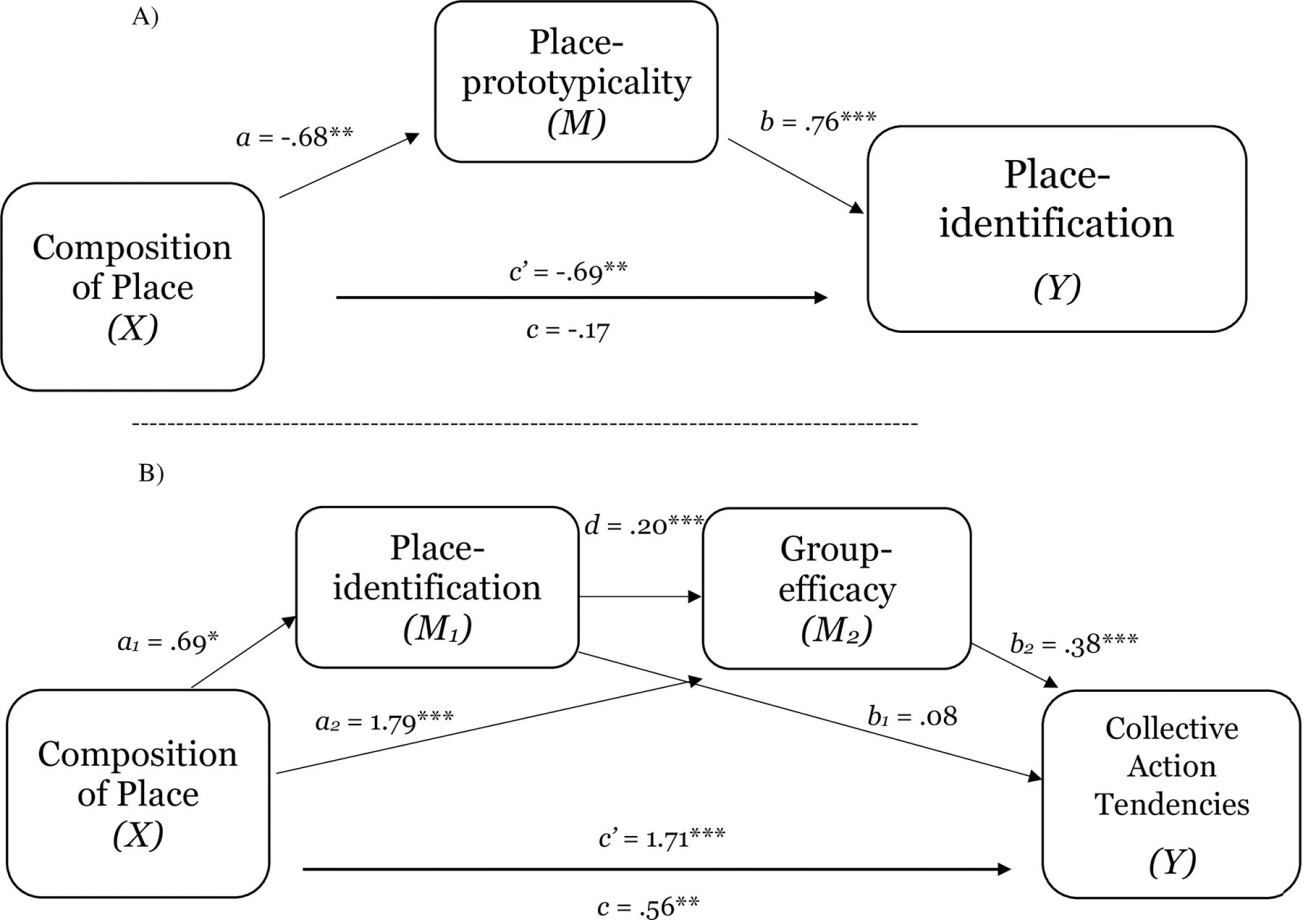
Test of the mediating effect of place-prototypicality on the relation between composition of place and place-identification in study 4c (Panel A). Test of the serial mediating effect of place-identification and group efficacy in the relation between composition of place and collective action tendencies in study 4c (Panel B). Notes: *p < .05, **p < .01, ***p < .001.

Consistent with the previous studies, composition of place had a direct effect on perceptions of place-prototypicality, *t*(148) = 2.39, *p* < .05, *d* = 0.38, 95% CI = [.11, .66],and place-identification, *t*(105) = -2.53, *p* < .05, *d* = 0.40, 95% CI = [.13, .68]. Under conditions of higher group-representation (*M*_**PP**_ = 4.62, *SD* = 1.86; *M*_**PI**_ = 5.14, *SD* = 1.75), participants were more likely to perceive the University as prototypical of their group and report sense of belonging, compared to under conditions of relatively lower group-representation (*M*_**PP**_ = 3.94, *SD* = 1.64; *M*_**PI**_ = 4.44, *SD* = 1.70). Replicating the past studies, place-prototypicality explained the relation between composition of place and place-identification (see Table 5 and Fig 13, Panel A).

There was also an effect of composition of place on group-efficacy, *t*(148) = 8.65, *p* < .001, *d* = 1.50, 95% CI = [1.31, 1.72] and collective action tendencies, *t*(148) = 6.05, *p* < .05, *d* = 0.89, 95% CI = [.68, 1.13]. Under conditions of higher group-representation (*M*_**GE**_ = 5.54, *SD* = 1.37; *M*_**CA**_ = 5.35, *SD* = 1.54), participants reported higher group-based efficacy and willingness to act on behalf of their group, compared to under conditions of relatively lower group-representation (*M*_**GE**_ = 3.60, *SD* = 1.20; *M*_**CA**_ = 4.07, *SD* = 1.30). Consistent with the notion that social support is associated with group efficacy [96], results demonstrate that sense of belonging (place-identification) and group efficacy (sequential mediation) explained the effect of composition of place on collective action tendencies (estimate .07, *SE* .04; 95% CI = .01 to .12; see Table 5 and Fig 13, Panel B).

The results illustrate that composition of place has a direct effect on a group member’s willingness to engage in collective action. Although both conditions suggested minority-group status, minority group members reported greater group-based efficacy and willingness to take action under conditions of relatively greater group representation. Moreover, the current findings suggest that place-identification (sense of belonging) may serve as an antecedent to group-efficacy, one of the ‘core’ motivations for collective action [99]. Whereas much of the work on the antecedents to collective action has focused on individual- and group-level factors, such as group-identification, anger, and moral conviction [99], the current work suggests *how* these antecedents manifest may be dependent on level of group representation and place-identification. The final study was designed to extend the current work by examining whether composition of place affects an additional indicator of propensity to view place in group-based terms.

## Study 5a: Level of group representation within place and labeling place in terms of social identity

To provide evidence with greater external validity regarding the effect of composition of place on propensity to view place in group-based terms, Study 5a used a quasi-experimental design in a classroom setting. More specifically, data was collected in a classroom (the ‘place’ of investigation) and included a new measure of viewing place in group-based terms (i.e., likelihood of seeing place in terms of specific social identities; e.g., “Male space” or “Female space”).

### Study 5a

#### Method

One hundred and forty-six participants (99 women and 47 men; *M* age = 22.96) were recruited from two large sections of introductory psychology courses (n = 250 for each course) in the first week of each course. The sample consisted individuals that self-identified with a variety of racial/ethnic groups: White (n = 49), Hispanic/Latinx (n = 46), Black/African-American (n = 37), Asian/Asian-American (n = 9), and bi-racial/mixed (n = 5). Consistent with national trends in the field of Psychology (U.S. Department of Education, 2018); both sections had a clear majority of self-identified women, compared to men (~70% of the classroom sections were women). Thus, for the purposes of this study, women (n = 99) were the numerical majority group (high group representation within place) and men (n = 47) were the numerical minority group (low group representation within place). All participants were asked to ‘consider the physical space of the classroom…including the look of the class, people in the room, and objects around the room.” Thus, all participants completed the survey in the actual place of the classroom. Next, utilizing the same items as the previous studies adapted to gender, place-prototypicality (*α* = .92) and place-identification (*α* = .86) were measured. In addition, two separate items assessed propensity to view the classroom in terms of social identity. Specially, the items asked participants the extent to which they would label the classroom as ‘male space’ versus ‘female space’ on a 1 (*not at all*) to 7 (*very much*) scale.

#### Results and discussion

Composition of place had a direct effect on group members’ perceptions of the group-prototypicality of place, *t*(144) = 15.59, *p* < .001, *d* = 2.83, 95% CI = [2.65, 2.96] and identification with place, *t*(144) = 4.03, *p* < .001, *d* = 0.72, 95% CI = [.50, .55]. Women, with greater group-representation in the classroom, were more likely to perceive the physical classroom as prototypical of their group and identify with the classroom (*M*_**PP**_ = 4.82, *SD* = 0.97; *M*_**PI**_ = 3.59, *SD* = 1.37), compared to men, that had low group representation in the classroom (*M*_**PP**_ = 2.19, *SD* = 0.88; *M*_**PI**_ = 2.63, *SD* = 1.29). There were no differences between men and women in their likelihood to label the classroom as ‘male space’, *t*(144) = 1.91, *p* = 05, men were more likely to label the class as ‘female space’ (*M* = 6.26, *SD* = 0.64), compared to women (*M* = 5.35, *SD* = 1.30), *t*(144) = -4.48, *p* < .001, *d* = -0.88, 95% CI = [-.98, -.61]. The results provide additional evidence within an ecologically valid place of how level of group representation shapes propensity to view place in group-based terms using two indicators, as well as sense of belonging.

## General discussion

Why does group representation matter? The present work speaks to this fundamental question by providing robust evidence linking the social make-up of a local place (i.e., level of group representation or composition of place), perceptions of the group-based nature of place, sense of belonging, and collective behavioral tendencies. Across a diverse set of groups (race/ethnicity; gender; religion) and places/contexts (classroom; organization; university, restaurant; law-enforcement; nation), the findings provide support not only for the integral role of composition of place for understanding collective behavior (i.e., there are distinct ways of experiencing and acting in place as function of group representation), but also for a group-based approach to place. First, composition of place is directly associated with propensity to view places in group-based terms via perceptions of place-prototypicality and labeling place in terms of social identity (e.g., “White space” or “Black space”). A group-neutral approach to place assumes that people perceive, approach, and understand places as being not representative or characteristic of a particular group (or groups). The current findings suggest, however, one should not assume all people or groups in a local place view the respective place in group-neutral terms, nor that similar places are perceived the same in terms of group-based perceptions. Second, the present work illustrates that perceptions of place-prototypicality is an antecedent to sense of belonging. Third, the social make-up of place in terms of numeric group representation shapes a comprehensive set of collective and intergroup outcomes. Rooted in distinct psychological contextualized experience as a function of minority versus majority-group status, level of group representation shapes a group member’s expectations, understanding, and behavioral tendencies.

Indeed, the effects of composition of place on numerous and a diverse set of outcomes speak to the comprehensive way in which composition of place shapes a group member’s experience.

The present work extends the literature in three central ways. First, the findings illustrate the direct effect of level of group representation within place on numerous and a diverse set of outcomes. These findings contribute to an emerging literature on the effects of numerical group representation and bolster the case for a ‘*minority-majority*’ distinction in the study of group behavior within specific contexts. Second, whereas much of the work within the social identity literature exploring prototypicality has focused on leaders and leadership [18], the current work introduces the construct of *place-prototypicality* and demonstrates its potential for explaining collective and intergroup outcomes. Third, a SPACE approach provides an organizing framework for the study of how characteristics of place shape group-member behavior. I elaborate on these contributions in the next sections.

### Level of group-representation & a distinct psychology of numerical group-status within place

Across several studies, the current work provides clear evidence that group members with differing levels of representation within place have distinct contextualized experience within the respective place, which (in turn) explains unique patterns of collective and intergroup behavior (expectations, understanding, and behavioral tendencies). Indeed, moving from low (i.e., minority numerical group status) to high (i.e., majority numerical group status) group representation within place shapes how a person defines themselves (*self-categorization*), their propensity to view place as representing the unique beliefs or values of their respective group (*place-prototypicality*), and a person’s sense of psychological connection to the respective place (*space-identification*). Importantly, the effects of numerical status in place were consistent and reliable for both socio-national minority (e.g., Black/African-American participants) and majority (e.g., White participants) groups. That is, the findings generalized across groups of differing national numerical-group status for both race/ethnicity and gender, demonstrating the effects are context-driven and fundamentally about the social makeup of local place. Of course national numerical group status and group status within local place often co-occur, but the evidence suggests that group-status within place and not membership in a particular socio-demographic group *per se* (e.g., being a Black/African-American person) explain the distinct collective behavioral tendencies.

The results contribute to a wealth of empirical evidence that points to the conclusion that minority and majority-group status within place is associated with distinct psychological orientations. Indeed, numerical group representation within place, as manifested by minority or majority-group status, directly affects sense of belonging [100], cognitive orientation [101], anxiety [102], propensity to feel under a ‘spotlight’ [103], academic motivation [104], sense of threat in performance situations [e.g., stereotype threat; 33], trust [14], and stereotyping [105]—to name just a few. Taken together with the findings in the present work, there is a wealth of evidence illustrating distinct psychological experience and dissimilar realities based on numerical minority and majority-group status within place. This conclusion is bolstered by replication across a diverse set of literatures, such as neuroscience [106,107], social cognition [108], persuasion [109], health/well-being [110], education [111], and business [112]. The large body of work suggests that the ‘*minority-majority*’ group-status classification is an important, necessary, and meaningful distinction for studying and understanding psychological experience within social contexts and a useful means to organize and explain findings from across psychology.

Importantly, the present work suggests not just that minority and majority group members have distinct experience in place, but that there a host of benefits associated with majority-group status within a respective place. One of the most distinct differences between minority and majority-group members’ everyday experiences is the salience of place communicating ‘this place is for you’/‘not for you’ and who is *supposed* to be in a respective place. Given the large degree of segregation across most social, educational, and professional settings [e.g., race/ethnicity; religion; class; 113], the benefits of majority-group status are not merely theoretical, but have very real practical implications. Based on the current data, the results demonstrate that holding majority-group status within a respective place is associated with more positive expectations regarding group-based treatment, increased sense of belonging, less propensity to make attributions to discrimination, and greater expectation that fair procedures will be utilized to resolve disputes. Beyond the benefits associated with everyday experience, study 5a suggests that composition of place also shapes conception of places designed to be ‘group-neutral.’ What does it mean to have systematic differences between minority and majority group members’ sense that public institutions are prototypical of their group (i.e., represent the values of their group)? One additional benefit of majority-group status (at least at the national-level), therefore, is the general expectation that many public service entities, predominantly composed of majority-group members, are ‘for you.’ In sum, the luxury of majority-group status, whether based on national numerical status (e.g., people who identify as White; Heterosexuals) or context-driven (e.g., political orientation in disparate contexts), is that holding majority-group status fundamentally shapes the way one experiences social reality with a variety of benefits. The benefits of majority-group status, however, start with social identity-based contextualized experience. More generally, a critical piece of divergent experiences of place is associated with perceptions of group prototypicality.

### Place-prototypicality: Perceiving place as prototypical of your group

*Place-prototypicality* is defined as the extent to which a given place is perceived as being characteristic of a group, representing the unique values of the group, and exemplifying the beliefs that define the group. That is, the extent to which a place (or the characteristics of place) is representative of *the* factors that make the group distinct from other groups. Whereas prior theorizing on prototypicality has focused primarily on leader prototypicality [see 26,27 for reviews], the current work extends the study of group-prototypicality to place. Conceptually, it is expected that many of the positive benefits of leader prototypicality should translate to place-prototypicality, but also that place-prototypicality may help to explain a variety of other intergroup relations outcomes.

Consistent with work on leader prototypicality [27], places viewed as high in group-prototypicality should also be viewed as fairer, more trustworthy, and also lead to greater productivity. Thus, this work speaks to approaching the study of collective behavior by not only acknowledging that people can view places in group-based terms, but seeking to understand the implications of divergent understandings of the group-based nature of place.

More broadly, the current findings provide robust evidence that perceptions of place-prototypicality are an antecedent to sense of belonging, but also that both place-prototypicality and sense of belonging (together) shape collective behavioral tendencies, including comfort, intentions to stay in place, expectations of procedural justice, willingness to partner with law enforcement, and collective action tendencies. Beyond the investigation of place-prototypicality as an antecedent to sense of belonging and other positive outcomes, one intriguing avenue for future work is the exploration of place-prototypicality as a mediating construct.

The findings of study 3b, for example, provide initial evidence that concerns about group-prototypicality of place partially explain majority-group bias against a minority groups. More generally, these findings seem to suggest that place-prototypicality may be especially useful construct because group members have concerns about the ‘ideas or values of space or place’ That is, there is often a struggle for the predominant values of place and some intergroup conflict may be a result of groups vying for the ‘ideas of space or place.’ To the extent that conflict between liberals and conservatives, for example, is rooted in distinct moral values [114], it seems that some intergroup disagreements between the two political groups [e.g., “political correctness”; 115] is explained by each groups’ desire to have their group’s unique beliefs or values represented in spaces, places, and contexts across the national landscape (i.e., a desire for place-prototypicality). Thus, beyond the benefits of studying place-prototypicality as an antecedent to sense of belonging, the ‘ideas of place’ and the battle for who or which group should represent place is a compelling topic of future study.

#### Place-identification (sense of belonging) and collective behavior

Whereas much of the work on attachment to place or sense of belonging has been rooted in a sociological perspective [6], relatively little work has explored the implications of variation in identification with place as a function of group membership or social identity [cf. 116]. One of the central practical contributions of this work involves the evidence for the way identification with place can play a role in explaining collective behavior. Just as a wealth of empirical work demonstrates that high group identification can impact motivation [117], performance [118], or commitment to the group under threat [119], it would be expected that identification with place would play a similar role in shaping a variety of outcomes. Indeed, place-identification had a direct role in explaining several outcomes, including comfort, perceptions of competence of a target, expectations of procedural justice, and group-efficacy. Importantly, place-identification is pertinent to a variety of contexts, including education [e.g., classroom; academic performance; 120], health [e.g., a doctor’s office or hospital; patient health outcomes; 121], business [e.g., office; employee organizational commitment; 118], marketing [e.g., location-specific franchises; consumer behavior; 122], neighborhoods [e.g., town or city as space; gentrification; 123], or even sports [e.g., locker-room; team-building; 124]. As others have noted, identification with place is central to the nature of one’s experience in place and people’s understandings of ‘who belongs, who has rights, and who one feels a sense of commonality with’ [116]. Thus, one contribution of the present work is illuminating the role of identification with place in explaining collective and intergroup relations phenomena. More broadly, a SPACE framework provides a means for linking characteristics of place, social identity-based contextualized experience, and collective or intergroup behavior.

#### Social identity paradigm for contextualized experience (SPACE) as an organizing framework for studying collective and intergroup behavior

A *S**ocial identity* *Pa**radigm for* *C**ontextualized* *E**xperience* (SPACE) provides a framework for understanding how the characteristics of place inform social identity-based contextualized experience, which (in turn) organize how group members approach, understand, and behave within the respective place. The present work focused on one characteristic of place, composition or level of group representation, but there are variety of characteristics of place that cumulatively shape contextualized experience and (in turn) collective behavior. Moreover, although the present work primarily focused on racial/ethnic, gender, and religious group memberships, it would be expected to be relevant to any social identity or group membership within a given context (e.g., sexual orientation; class; ethnic groups).

A SPACE framework and group-based approach to place opens up opportunities to study a variety of collective and intergroup behaviors. Each place could be conceived as an ‘eco-system of characteristics’ that determine group members’ likelihood of viewing place in group-based terms, as well as their social-identity based contextualized experience. For example, demographic characteristics of leader [125], leadership style [126], norms [127], ideological climate [128], or physical objects in place [13] all likely play a role in shaping perceptions of the group-based nature of place and (subsequently) group member behavior. Thus, one potentially fruitful area of work is to explore how other characteristics of place directly shape collective and intergroup behavior, as explained by perceptions of place-prototypicality and sense of belonging (place-identification). The collection of findings also demonstrate the need to prioritize context and the place *of* collective and intergroup behavior in the development of theory, empirical methods, and interpretation of findings.

#### Place as a unit of analysis

The present studies complement recent work that seeks to shift away from a decontextualized individual-individual approach to collective behavior toward an approach that is rooted in specific context of intergroup relations [e.g., prejudice; 129,130]. Indeed, this approach suggests that to change individual, group, or intergroup relations behavior, interventions should focus on changing macro-level (rather than individual-level) social factors related to the situation or context. Consistent with this approach, a SPACE framework implies the need to devote much greater attention to *situational affordances and constraints* as a function of contextualized experience. Indeed, utilizing place as a unit of analysis would suggest a shift away from examining group differences based solely on individual-characteristics [e.g., perseverance; 131] to a framework that examines the advantages and disadvantages of place for the individuals, group members, and groups within the respective place. Thus, a group-based approach to place would suggest that research more fully explore how group-level differences in individual-level behavior may emerge because of affordances and constraints of a respective place (as a function dissimilar contextualized experience----arising from characteristics of the place and context).

Conceiving of place as a unit of analysis also has implications for the way practitioners make comparisons and the interpretation of findings. Scientists and practitioners often have a goal of ‘objective’ comparison between individual targets, but individual-level comparison requires (or assumes) equivalence of context-level factors. Integrating place and contextualized experience in comparative analysis, therefore, challenges the logic of using individual-level comparisons. For example, with respect to the study of educational outcomes, the present results would suggest distinct social identity-based contextualized experience for people as a function of level of group representation in a class or school. Thus, students in different classes (i.e., between-comparison; e.g., Black/African-American students within a predominantly White classroom compared to Black/African-American students in a racially integrated classroom), but also students in the *same* class (i.e., within-comparison; e.g., Black/African-American and White students in a single predominantly White classroom) likely have very dissimilar social identity-based contextualized experiences, which likely (at least partially) affects their performance [see sense of belonging work; 120]. An individual-level analysis would fail to account for the potential role of composition of place and social identity-based contextualized experience on educational outcomes for these respective students. Indeed, the collection of results suggest acknowledging (at minimum) or incorporating (at best) the place or context of a study in analyses and interpretation of findings will produce more nuanced findings and better science. Thus, it seems incomplete, for example, to say “Black/African-American” and/or “White” people act in a particular ways. More accurately, particularly if the goal of the research concerns making more generalizable comparisons, which replicate across contexts [i.e., moving beyond the replication crisis; 132 Klein, Ratliff, Vianello, Adams, et al., 2014], one might say “Black/African-American and White individuals within predominantly White contexts,” “Black/African-American and White individuals within predominantly Black/African-American contexts,” or “Black/African-American and White individuals within integrated contexts.” In sum, rather than divorcing ourselves from the social context of the participants and study of our work, the results clearly demonstrate the need to more fully engage in the ‘social’ of the social psychology of intergroup relations [133], especially relative level of group representation, which will allow for a more nuanced study of human behavior. The strong effect of composition of place on a variety of outcomes also has implications for diversity science.

#### Diversity science & a contextualized-experience driven account of how to create ‘inclusive’ space

As nations become more diverse, many cities/neighborhoods, businesses, and universities are concerned with the question of how to create an inclusive environment in the context of diverse populations. The findings of the present work have implications for diversity science and speak to the need for a more nuanced understanding of what constitutes a ‘diverse’ place or space. Much of the focus on diversity initiatives emphasizes representation [134]. Complimenting this work, the current findings clearly speak to the importance of group-based representation. However, the collection of studies also reveal that the underlying mechanism of the effects of representation is the sense that a place is characteristic of the unique characteristics that define one’s group (place-prototypicality) and sense of belonging or identification with place. Thus, the mechanisms explaining the effects of group representation on collective outcomes, clearly suggests there is an importance in exploring group members’ subjective contextualized experience. As demographic composition of nations change [135] and majority groups begin to act more explicitly in terms of racial/ethnic group membership or identity [136], a dynamic approach to diversity is necessary [137]. Indeed, a definition of ‘diverse’ space or place should not only be rooted in numeric representation, but also be attuned to the social identity-based contextualized experiences (place-prototypicality and place-identification) of different groups. Do all people feel the place (at least partially) represents their group? Do all people feel connected to the place?.

Defining ‘diverse space’ based on social identity-based contextualized experience of all group members suggests there is not one solution to issues related to diversity, but solution(*s*) linked to the specific challenges and opportunities of respective contexts. Thus, a group-based approach to place would suggest that the goal of creating ‘diverse or inclusive’ space explicitly for improving intergroup relations should not necessarily be to create places that benefit one group (e.g., racial/ethnic minorities) over another group (e.g., Whites), but to create places that are responsive to both minority and majority perspectives and rooted in identity-safety [138]. To the extent that one might expect that any group that assumes majority-group status would likely show in-group favoritism and an assimilative orientation toward minority groups in the respective context [38], it becomes integral for those interested in diverse spaces to strive for a functional equilibrium. Indeed, much like the tragedy of the commons [139], as each group vies for majority-group status (either numerically or based on a predominant set of ideas), it diminishes the capacity of all other groups to connect to the respective place. Thus, this ‘diversity equilibrium’ would suggest that truly diverse spaces, with no group holding a majority, require a sacrifice or a ‘diversity bargain’ if you will—in which no one group feels maximally connected to the place, but no group(s) feel disconnected from the place. Of course, any ’diversity bargain’ would also require integrating power, status, and history of the respective groups in the local place. A diversity science, though, should be attuned to the group-based nature of place and explore the characteristics of place that maximally benefit the most groups to achieve healthy outcomes relevant to diversity, equity, and inclusion.

#### Limitations & future directions

It is worth noting limitations and areas for further investigation. First, the present work was concerned with numeric group representation of local place, but does not factor how other characteristics of local setting might shape social-identity based contextualized experience. Thus, it is theorized that the social-make up of a place in terms of numeric group representation is a foundational characteristic (i.e., other characteristics build upon composition of place), but a variety of social and physical features or cues may be associated with distinct social identity-based contextualized experience [e.g., 16]. Second, the present work primarily utilized m-turk samples and a cross-sectional design, which limit the conclusions that can be drawn regarding causal direction. Relatedly, these studies utilized a survey-design (i.e, asking about place, rather than studying place in-person) to examine place. Additional evidence replicating the current results within naturalistic settings or via longitudinal data would help strengthen the evidence in support of the specified hypotheses. Nevertheless, a large body of work suggests the quality of data obtained from online data collection is comparable to student and professional samples [140]. In addition, numerous studies in organizational psychology conducted in the field—including field studies in the present work—provide evidence complimenting the general pattern of results [141]. Third, the current studies used an expansive definition of place—encompassing a variety of geographies—but additional work is needed to further generalize this work to specific places. There is a large, diverse, and interdisciplinary literature on “place or space” [4–7,142], such that there are different notions of the way place is connected to space and dissimilar ways to operationalize ‘place.’ Whereas the consistent pattern of findings across the respective places in the current work can be viewed as strength—future work should seek to replicate these findings in specific places.

Fourth, several of the studies used manipulation-checks that may raise concerns about demand characteristics. That is, the studies included multifaceted descriptions of place in the respective studies, but the manipulation-check items may have cued participants to the purpose of the studies. Therefore, additional work is needed to examine the effects of composition of place using alternative methods. Fifth, the present work primarily focused on three facets of social-identity based contextualized experience (self-categorization, space-prototypicality, and space-identification), but there is room for further investigation around the interrelation between the three facets of contextualized experience, as well as potential moderators of the effects of composition of place on collective behaviors. Thus, although I would theorize that self-categorization is the proximal construct, which shapes the relevant social identity for perceptions of place-prototypicality and identification with place, the present work did not explore the interrelation among all three facets of social identity-based contextualized experience. Moreover, there is also a need to examine factors that moderate the effects of composition of place on group behavior at both the individual [e.g., group-identification; 143] and structural [e.g., perceived permeability of group boundaries; 144] level. Sixth, the current studies focused on numerical group representation, but failed to explore power. Although status and power can be positively associated, they are distinct constructs and thus one should avoid confounding the two constructs [145]. Indeed, numerical representation does not necessarily equate to power, such that if you put unequal parties within place, outcomes derived in the respective place often reflect the fundamental inequality of asymmetrical power relations outside of the place [146]. Thus, one avenue for future work concerns the extent to which power dynamics can moderate the effects of level of group representation within place on collective outcomes.

Finally, for ease of investigation, the present studies used broad racial/ethnic categories, collapsing across different types of racial/ethnic groups to classify both “majority” and “minority” group status, but additional work should explore nuances associated with distinct racial/ethnic groups. For example, although there is certainly a common experience rooted in minority status, there are important experiential distinctions not only among groups identifying in a single category (e.g., “Black/African-American”; “Latinx”), but also between different racial/ethnic/religious minority groups. Similarly, majority groups (e.g., Whites) differ based on a variety of demographic, cultural, and ideological factors, suggesting distinct behavioral patterns as a function of these potential moderators. Therefore, a more nuanced approach to the study of contextualized experience would not only explore the unique experience of multiple groups and multi-identity groups, but also dimensions of intersectionality (Crenshaw, 1997; i.e., systems of advantage or disadvantage are not singular).

## Conclusion

The present research provides evidence for the integral role of composition of place or level of group representation in shaping propensity to view place in group-based terms, sense of belonging, and a host of collective and intergroup outcomes. The relative level of group representation of place can shape our collective imaginations about a particular place. The current work suggests who inhabits place implicitly communicates group-relevant meaning, particularly about a set of predominant beliefs and values, as well as ‘who belongs’ and ‘who does not.’ Thus, who is and isn’t represented within place sets the foundation for the geography of social identity, collective behavior, and intergroup relations.

## Supporting information

S1 File (SAV)

S2 File (SAV)

S3 File (SAV)

S4 File (SAV)

S5 File (SAV)

S6 File (SAV)

S7 File (SAV)

S8 File (SAV)

S9 File (SAV)

S10 File (SAV)

S11 File (SAV)

S12 File (SAV)

S13 File (SAV)

S14 File (SAV)
